# Estimating the cost-effectiveness of salt reformulation and increasing access to leisure centres in England, with PRIMEtime CE model validation using the AdViSHE tool

**DOI:** 10.1186/s12913-019-4292-x

**Published:** 2019-07-16

**Authors:** Adam D. M. Briggs, Jane Wolstenholme, Peter Scarborough

**Affiliations:** 1 Centre on Population Approaches for Non-Communicable Disease Prevention, Nuffield Department of Population Health, University of Oxford, Old Road Campus, Headington, Oxford, OX3 7LF UK; 2 Health Economics Research Centre, Nuffield Department of Population Health, University of Oxford, Oxford, UK; 3 Centre on Population Approaches for Non-Communicable Disease Prevention and NIHR Biomedical Research Centre at Oxford, Nuffield Department of Population Health, University of Oxford, Oxford, UK

**Keywords:** Public health economics, Modelling, Economic modelling, Non-communicable disease, Diet, Physical activity, Public health

## Abstract

**Background:**

PRIMEtime CE is a multistate life table model that can directly compare the cost effectiveness of public health interventions affecting diet and physical activity levels, helping to inform decisions about how to spend finite resources. This paper estimates the costs and health outcomes in England of two scenarios: reformulating salt and expanding subsidised access to leisure centres. The results are used to help validate PRIMEtime CE, following the steps outlined in the Assessment of the Validation Status of Health-Economic decision models (AdViSHE) tool.

**Methods:**

The PRIMEtime CE model estimates the difference in quality adjusted life years (QALYs) and difference in NHS and social care costs of modelled interventions compared with doing nothing. The salt reformulation scenario models how salt consumption would change if food producers met the 2017 UK Food Standards Agency salt reformulation targets. The leisure centre scenario models change in physical activity levels if the Birmingham Be Active scheme (where swimming pools and gym access is free to residents during defined periods) was rolled out across England.

The AdViSHE tool was developed by health economic modellers and divides model validation into five parts: validation of the conceptual model, input data validation, validation of computerised model, operational validation, and other validation techniques. PRIMEtime CE is discussed in relation to each part.

**Results:**

Salt reformulation was dominant compared with doing nothing, and had a 10-year return on investment of £1.44 (£0.50 to £2.94) for every £1 spent. By contrast, over 10 years the Be Active expansion would cost £727,000 (£514,000 to £1,064,000) per QALY.

PRIMEtime CE has good face validity of its conceptual model and has robust input data. Cross-validation produces mixed results and shows the impact of model scope, input parameters, and model structure on cost-per-QALY estimates.

**Conclusions:**

This paper illustrates how PRIMEtime CE can be used to compare the cost-effectiveness of two different public health measures affecting diet and physical activity levels. The AdViSHE tool helps to validate PRIMEtime CE, identifies some of the key drivers of model estimates, and highlights the challenges of externally validating public health economic models against independent data.

**Electronic supplementary material:**

The online version of this article (10.1186/s12913-019-4292-x) contains supplementary material, which is available to authorized users.

## Background

The PRIMEtime CE model is a multistate life table model that can estimate and compare the cost-effectiveness of interventions affecting the distribution of dietary risk factors and physical activity levels in the population [1]. Approximately 38% of the non-communicable disease burden in England is thought to be attributable to modifiable risk factors, with poor diet and low levels of physical activity being two of the leading causes [2]. PRIMEtime CE aims to help public health decision makers in England choose where to spend limited resources and maximise health.

Models simplify reality in order to estimate and predict real world outcomes, and model validation aims to understand how accurately a given model represents reality. Validation can take many forms, and ideally model outputs should be prospectively compared with what subsequently transpires. However, this is a particular challenge for public health economic models predicting non-communicable disease (NCD) outcomes because of the nature of these outcomes. NCDs often take many years to develop and are influenced by a complex system of determinants where identifying the independent effect of a single intervention can be extremely challenging [3].

The importance of model validation is emphasised by how different health economic models within well-established fields such as a cancer, chronic obstructive pulmonary disease (COPD), and diabetes can lead to very different cost-effectiveness estimates of the same intervention [4–6]. There are different approaches to validating health economic and NCD models [7–9], with the recently published Assessment of the Validation Status of Health-Economic decision models (AdViSHE) tool providing a generic approach to validating health economic models that is applicable to public health interventions [9]. Furthermore, the AdViSHE tool can be useful for identifying where additional validation efforts may be helpful.

In this paper we use PRIMEtime CE to estimate the cost effectiveness of reformulating food in England such that it meets the 2017 Food Standards Agency (FSA) salt targets, and of expanding the Birmingham Be Active scheme, which provides free access to leisure centres for Birmingham City residents at certain times of the week. We then aim to validate these results using the AdViSHE tool [9].

## Methods

### PRIMEtime CE

PRIMEtime CE is a multistate life table model that uses routinely available data to estimate and compare the cost-effectiveness of interventions affecting one or more of thirteen dietary risk factors and physical activity levels in the English population. The model estimates the age and sex-specific impact of an intervention on a closed adult cohort by changing the distribution of the risk factor in the population and quantifying the subsequent effect on population mortality and morbidity. Modelled diseases are ischaemic heart disease (IHD), stroke, type two diabetes, seven cancer subtypes (breast, lung, colorectal, stomach, liver, kidney, and pancreas), and liver cirrhosis. All model parameters are estimated using meta-analyses of randomised controlled trials or prospective cohort studies. The impact on disease of a change in risk factor is either modelled directly or via one of three intermediate risk factors: body mass index (BMI), blood pressure, and serum blood cholesterol. Disease costs are derived from NHS programme budgeting costs (a disaggregation of total NHS England expenditure by ICD-10 code), and social care costs are taken the Personal Social Services Research Unit costs for monthly local authority residential care in England and are calculated as a function of age and quality of life. Utility decrements are from Sullivan et al., a catalogue of EQ-5D utility values covering 135 ICD-9 codes [10–12]. PRIMEtime CE also includes estimates of the costs and morbidity associated with unmodelled diseases that accrue as people age.

The primary outcome is cost-effectiveness over a 10-year time horizon - or return on investment in circumstances where the intervention is cost-saving - from a health and social care perspective in England. The time horizon and economic perspective where chosen following stakeholder feedback (see Briggs et al. [1]). In the primary analysis, costs to government and industry are included, with all costs and health outcomes discounted at 1.5%. Cost-effectiveness is: (C_b_ – C_a_) / (E_b_ – E_a_); C_b_ = sum of 10-year intervention costs and expenditure on health and social care; C_a_ = 10-year health and social care costs with no intervention; E_b_ = total QALYs in 10 years following the intervention; E_a_ = 10-year QALYs with no intervention. In the case where (C_b_ – C_a_) is negative, return on investment is calculated as: (C_b_ – C_a_)/C_i_; C_i_ = cost of the intervention. No direct economic value is attributed to QALYs. The potential impact of using different model parameters is explored with a range of sensitivity analyses.

A detailed description of the model, including model parameters, can be found in the manuscript describing the model [1].

### Modelled interventions

Interventions modelled are reformulating food to have less salt [13], and expanding the Be Active scheme - providing over 1 million residents of Birmingham City free access to local leisure centres at certain times of the day - across the whole of England [14]. These two interventions were chosen to be modelled by stakeholders including charities, government officials, and patients who were asked to select one physical activity intervention and one dietary intervention from a list of eight policy options. The options presented to stakeholders were selected based on there being data available with which to parameterise the relationship between the intervention and the change in risk factor (diet or level of physical activity), and on the intervention either being recommended by NICE public health guidance or of stated importance to the UK government in 2014. All data relate to the English population in 2014 unless otherwise stated.

### Salt reformulation

The salt reformulation intervention estimates the impact on stroke and IHD of achieving the FSA ‘at home’ 2017 salt targets for all food consumed, with a baseline year of 2014 [13]. Salt consumption in England was taken from the combined results of four years of the National Diet and Nutrition Survey (NDNS) from between 2008 and 2012 and assumed to be unchanged between then and 2014 (more recent NDNS results from 2013 and 2014 were not available at the individual level at the time of the analysis) [15]. For each of the food groups in NDNS, the average salt content was changed to the FSA 2017 target to calculate what the age and sex-specific salt consumption would be following the intervention, assuming there is no other change in the individual’s diet. The standard error of the difference in salt consumption between baseline and following the intervention was calculated from NDNS data for each age and sex group and used to estimate uncertainty in the intervention’s effect size, assuming a normal distribution.

Both industry and government costs were estimated. Industry costs are the costs required to reformulate food and were taken from Collins et al. who estimated the cost-effectiveness of different policies aimed at reducing salt consumption and CHD in England [16]. Government costs were split into monitoring costs and administrative costs. Monitoring costs were estimated based on the current costs to Public Health England of urinary sodium surveys taking place every other year to monitor salt consumption and Kantar Worldpanel purchasing data for foods to monitor their salt content. In 2014, the urinary sodium survey in England cost £327,289, and the annual cost of the Kantar dataset in 2016 was £94,100 (£91,588 in 2014 prices) [17]. Finally, Government administrative costs were calculated using the WHO costing tool for prevention and control of NCDs (WHO NCD costing tool) following methods described by Webb et al. [18, 19]

The intervention was assumed to be introduced evenly over three years between 2014 and 2017, with a third of the salt reduction and the industry costs occurring in each year. Full implementation in year one was explored as a sensitivity analysis. More detail on how the salt reformulation intervention was modelled, and how costs and their uncertainty were calculated, can be found in pages two to five of the Additional file 1.

### Expanding the Birmingham Be Active scheme

Pre-intervention (baseline) physical activity levels for adults aged over 16 years by five-year age group and by sex in England were taken from the Active People Survey, 2010–2011 (APS) [20]. The APS surveys a representative English population sample to understand participation rates in a range of activities such as walking and cycling, mirroring the questions asked in the Be Active economic evaluation. The population was divided into four categories: sedentary (zero minutes of moderate physical activity per week), under active (less than 60 min of moderate physical activity per week but not sedentary), active (60–150 min per week), and recommended (meeting the UK Chief Medical Officers’ recommendations of 150 min of moderate physical activity per week or more) [21].

Data from a health-economic evaluation of Be Active by Frew et al. was used to estimate the impact on population physical activity levels and on costs of expanding the Be Active scheme to all residents in England [14]. Frew et al. estimated the proportion of residents aged over 16 years participating in Be Active and their pre- and post-intervention levels of physical activity, divided into three categories: under active, active, and recommended (by definition, none of those participating were sedentary).

Following the intervention, those enrolled in the programme changed physical activity category based on data from Frew et al. These results were then used by PRIMEtime CE to estimate the impact on IHD, stroke, type two diabetes, breast cancer, and colorectal cancer. Uncertainty estimates for all input parameters and data are included in the modelling. A detailed description of the modelling of expanding the Be Active scheme can be found in pages six to 10 of the Additional file 1.

### Sensitivity analyses

Sensitivity analyses explore the effects on cost-effectiveness of changing PRIMEtime CE’s principle assumptions including time horizon, discount rate, and removing industry costs (see Additional file 1: Table S1).

### Model validation

The AdViSHE tool was developed by a panel of expert health economic modellers and decision makers to help model developers prioritise validation efforts. The tool divides validation into five parts:

A. validation of the conceptual model;
B. input data validation;
C. validation of the computerised model;
D. operational validation; and
E. other validation techniques.

In this paper, we discuss how PRIMEtime CE performs against each of these five parts using results from the two modelled interventions.

## Results

### Salt reformulation results

Reformulating food to meet the 2017 salt targets was estimated to reduce salt consumption among those in England aged 15 years and above by an average of 1.0 g salt (409 mg sodium) per day (1.2 g [477 mg] among men, 0.9 g [344 mg] among women). These declines in salt consumption would lead to an average reduction in systolic blood pressure of 1.2 mmHg among men and 0.8 mmHg among women. For both men and women, the largest falls in salt consumption and blood pressure were among 18–34 year olds. Baseline and post-intervention salt consumption by age and sex is shown in Additional file 1: Table S2.

Total industry costs included in PRIMEtime CE were £599 m (20,000 products reformulated at £29,953 per product - see pages three to four of the Additional file 1 for more details). Annual government monitoring costs were estimated to be £255,233 based on annual Kantar data for monitoring salt in different food products and for a biannual urinary sodium survey; annual government administration costs were £570,892 per year.

**Fig. 1:**
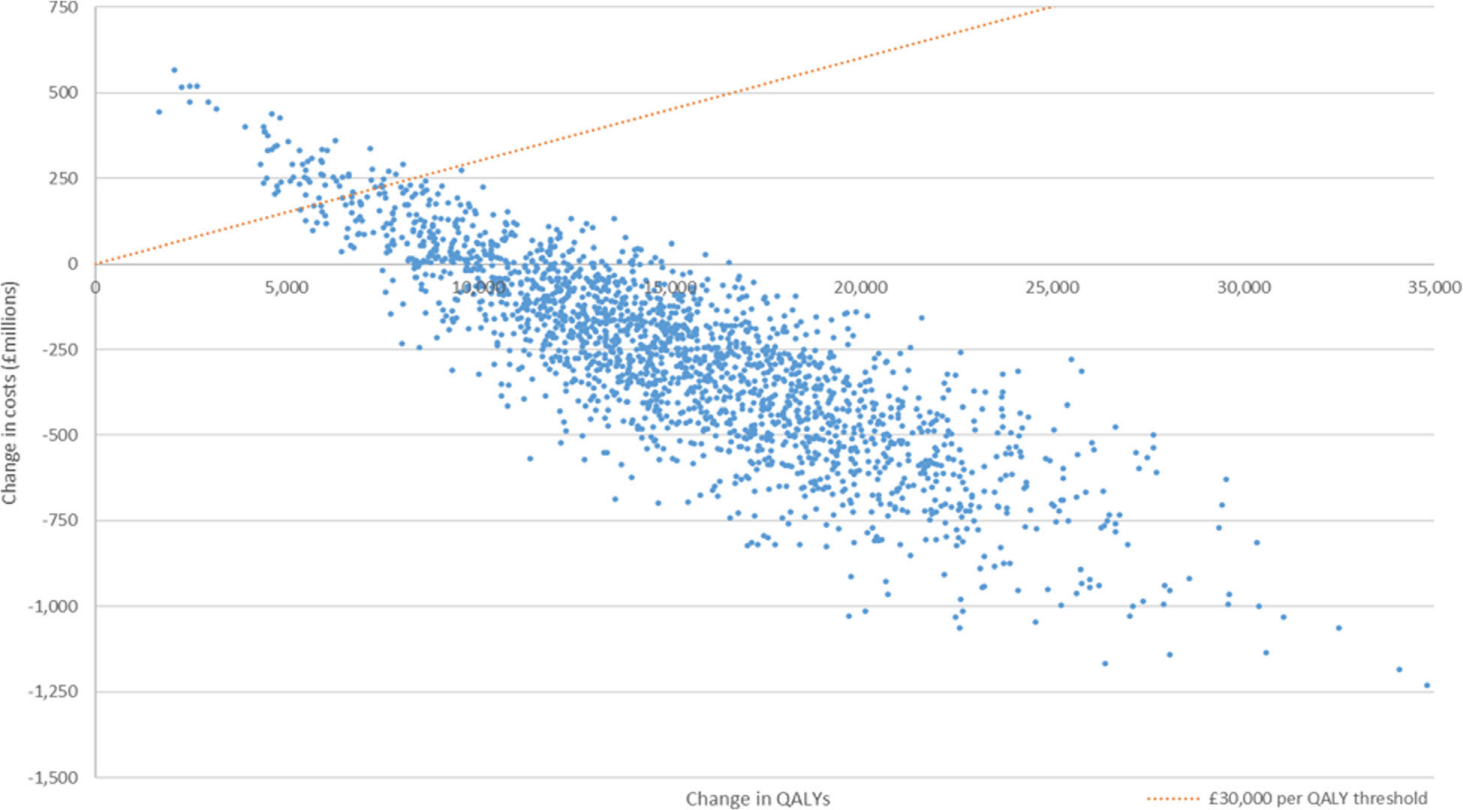
Cost effectiveness plane showing results of the salt intervention. QALY, quality adjusted life year

Over 10 years, the intervention resulted in a median gain of 15,000 QALYS (95% UI 5930 to 25,520), an NHS cost saving of £141.7 m (£58.8 m to £235.9 m), and a saving from social care expenditure of £724.8 m (£306.5 m to £1200 m) at a total implementation cost to industry and government of £598.0 m (£489.2 m to £717.7 m). The intervention was dominant compared with doing nothing, with a return on investment (including NHS costs, social care costs, industry implementation costs, and government implementation costs) of £1.44 (£0.50 to £2.94; mean return on investment, £1.47) for every £1 spent, and a 97% chance of falling below the £30,000 upper limit cost effectiveness threshold used by NICE [22]. This does not include attributing a direct economic value to QALYs which would further increase the return on investment. The intervention became cost saving nine years following its implementation, after which time NHS and social care savings outweighed industry and government costs. Table 1 shows the results of the intervention by sex, and Fig. 1 illustrates the 2000 iterations of the Monte Carlo analysis on the cost-effectiveness plane.

**Table 1:** Summary of results of salt reformulation intervention

	Males (95% CI)	Females (95% CI)	Total (mean; 95% CIs)
Cost per QALY – overall (£)*	–	–	Dominant
Return on investment (£ for £1 spent)*	–	–	1.44 (1.47; 0.50 to 2.94)
Change in QALYs	9045 (3629 to 15,338)	5945 (2320 to 10,186)	15,000 (15,275; 5929 to 25,519)
NHS savings (£)	95,623,517 (39,784,418 to 159,346,380)	45,721,114 (18,519,937 to 76,690,196)	141,701,342 (141,921,578; 58,825,774 to 235,925,461)
Social care savings (£)	350,748,015 (148,153,324 to 582,543,734)	371,065,063 (154,269,323 to 619,047,856)	724,832,212 (730,558,705; 306,525,399 to 1200,201,282)
Intervention costs (£)*	598,039,731 (489,209,385 to 717,730,482)	598,039,731 (489,209,385 to 717,730,482)	598,039,731 (599,844,427; 489,209,385 to 717,730,482)

*The intervention and its costs affect the entire population and cannot be targeted such that only one subgroup is affected. Therefore, cost per QALY results are not reported by sex. Results presented are the median values from 2000 iterations of a Monte Carlo simulation, as such, the numbers in the final column may not equal the sum of males and females; 95% uncertainty intervals in parentheses; QALY, quality adjusted life year

The intervention led to 24,460 (10,573 to 38,686) fewer cases of stroke and 16,570 (7170 to 26,220) fewer cases of IHD over 10 years (Additional file 1: Table S3). There were small increases in other modelled diseases due to increased life expectancy following the intervention, allowing more time for other diseases to develop. Results varied by age and sex. Men had greater health benefits per person, and in total, than women, and greater NHS and social care savings per person. Women were responsible for more social care savings due to living longer and therefore having longer to accrue savings resulting from having a higher quality of life (and therefore lower social care costs) following the intervention. The greatest gain in QALYs per person was among men aged 70–74 years at the start of the intervention, with an average of 126 QALYs gained per 100,000 men. Social care savings peaked at £134 per person among men aged 75–79 years, and NHS savings per person peaked at £10 per person for men aged 65–69 years. Aggregate change in QALYs and costs are shown in Additional file 1: Figs. S1 and S2.

### Expanding the Be Active scheme results

The percentage of men achieving recommended levels of physical activity following the expansion of the Be Active scheme across England was estimated to increase from 46.4 to 47.7% alongside a decrease among under active men from 17.4 to 16.6%. Among women, there was a slightly higher absolute and relative increase in the percentage achieving recommended levels of physical activity (38.6 to 40.0%) with an associated decrease in under active women from 19.4 to 18.6%.

Younger adults had lower percentages of sedentary individuals and higher percentages of individuals achieving recommended levels of physical activity than older adults, with the same pattern seen for men compared with women. As a result of higher percentages of younger people and women enrolling in the intervention, the absolute percentage gains in those achieving recommended physical activity levels were higher in these population groups (see Additional file 1: Table S4 for results by age and sex).

Intervention costs per participant in 2014 after adjusting for inflation were £53.80 (triangular distribution 43.50, 53.80, 85.90) in year one, and £29.80 (24.00, 29.80, 48.10) in year two onwards. Total intervention costs were therefore estimated to be £202.6 m (163.8, 202.6, and 323.3) in year one and £112.1 m (90.5, 112.1, 181.1) in year two onwards. Per participant costs were included in PRIMEtime CE with triangular distributions used to quantify uncertainty in these estimates, as used by Frew et al. [14].

**Fig. 2:**
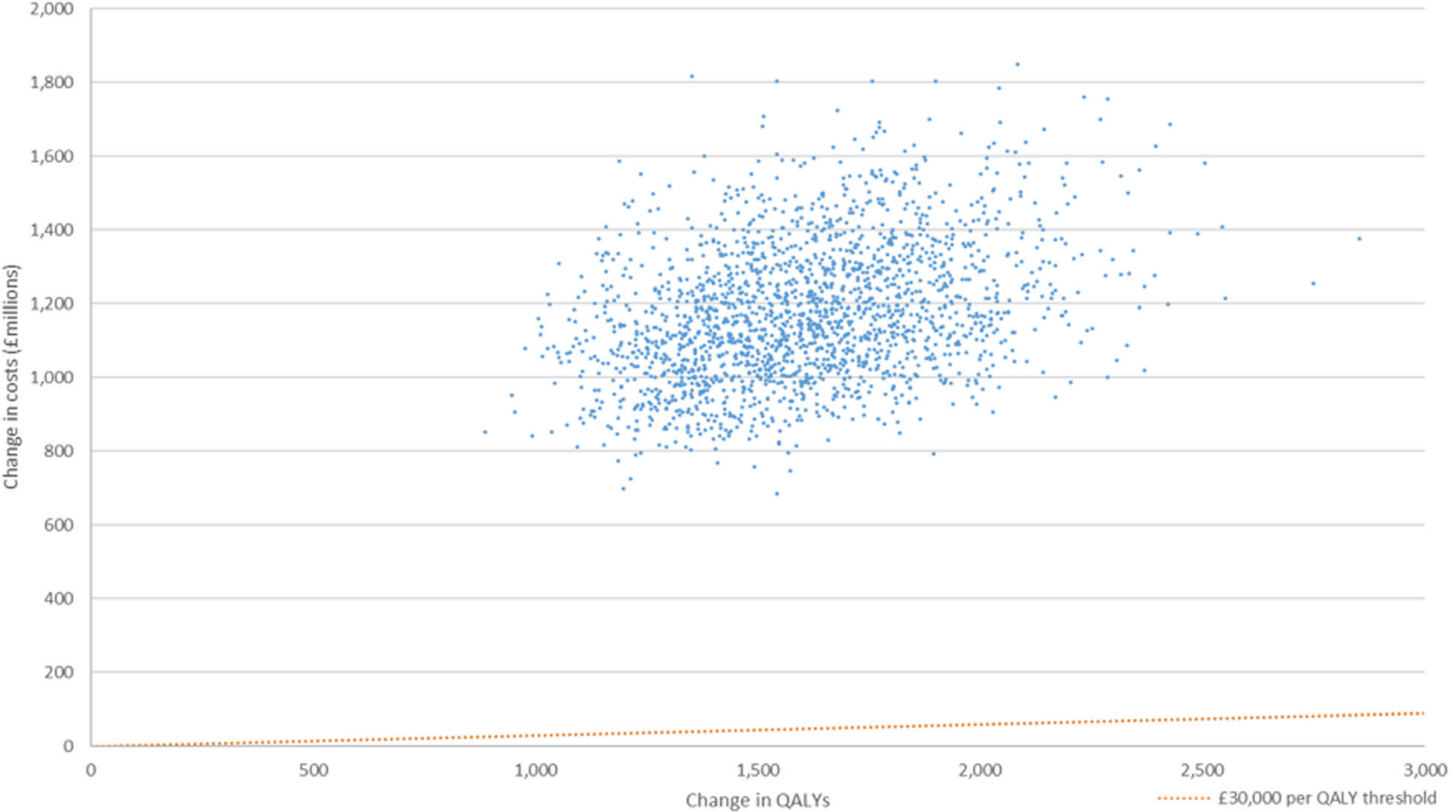
Cost effectiveness plane showing results of expanding the Be Active Scheme. QALY, quality adjusted life year

The Be Active expansion resulted in a median increase of 1600 (1130 to 2170) QALYs over 10 years with savings to the NHS of £10.40 m (£7.19 m to £14.58 m) and social care of £14.18 m (£7.84 m to £22.41 m). The intervention cost £1177 m (£882 m to £1602 m) meaning the median cost per QALY was £727,000 (£514,000 to £1,064,000; mean cost per QALY, £743,000) compared with doing nothing; there was no probability of the intervention being below £30,000 per QALY (Table 2, Fig. 2). To achieve a cost per QALY of £30,000, 10-year intervention costs would need to be £72.44 m, approximately £2.20 per participant per year compared to the modelled costs of £53.80 per participant in year one and £29.80 in year two onwards.

**Table 2:** Summary of results following the expansion of the Be Active scheme

	Males (95% CI)	Females (95% CI)	Total (mean; 95% CI)
Cost per QALY - overall	652,388 (458,290 to 951,568)	818,121 (580,315 to 1,186,274)	727,300 (743,301; 513,736 to 1,063,585)
Change in QALYs	872 (614 to 1197)	723 (518 to 984)	1597 (1609; 1134 to 2169)
NHS savings (£)	5,772,494 (3,975,940 to 8,107,746)	4,627,015 (3,206,297 to 6,477,924)	10,402,110 (10,475,147; 7,190,945 to 14,583,876)
Social care savings (£)	6,997,737 (3,810,312 to 11,073,841)	7,186,580 (3,995,764 to 11,333,750)	14,181,040 (14,433,928; 7,838,893 to 22,409,996)
Intervention costs (£)	576,601,063 (431,869,562 to 784,540,463)	600,728,881 (449,926,458 to 817,269,990)	1177,329,943 (1197,898,500; 881,796,020 to 1,601,810,453)
Cost per QALY - NHS perspective (£)	660,208 (466,633 to 959,372)	829,675 (589,731 to 1,196,543)	735,851 (752,329; 521,112 to 1,071,650)
Cost per QALY - social care perspective (£)	658,986 (464,292 to 958,713)	824,804 (586,489 to 1,193,704)	733,402 (749,844; 519,764 to 1,069,979)

Results presented are the median values from the Monte Carlo simulation, as such, the numbers in the final column may not equal the sum of males and females; 95% uncertainty intervals in parentheses; *QALY* quality adjusted life year

The disease with the largest reduction in cumulative incidence was diabetes with 4200 (2970 to 5740) fewer cases over the 10 years of the intervention, followed by stroke (550 [300 to 870] fewer cases), IHD (210 [150 to 280]), colorectal cancer (130 [−60 to 400]), and breast cancer (130 [30 to 250]). Men experienced larger health benefits than women for all conditions except breast cancer (Additional file 1: Table S5). The largest average gain in QALYs was among men aged 40–44 years (5.6 additional QALYs per 100,000 people), and the greatest cost savings were among men aged 50–54 years for NHS costs (£0.41 per person) and men aged 75–79 years for social care costs (£2.72 per person). The overall cost per QALY ranged from £5.60 m for 15–19 year olds to £106,000 for 85–89 year olds. The intervention does not become cost-saving over the life-time of the cohort meaning there is no time to return on investment. See Additional file 1: Figures S3, S4 and S5 for detailed results by age and sex.

### Sensitivity and uncertainty analyses

Sensitivity analyses and discussion of the sources of model uncertainty (including tornado plots) are presented in the additional results section on pages 11 and 12 of the Additional file 1. Of all the sensitivity analyses, both interventions were most affected by changing the time horizon. Removing costs to industry also significantly increased the return on investment following salt reformulation. Other sensitivity analyses had less of an impact on the final cost per QALY.

### Model validation

#### Part a: validation of the conceptual model

The AdViSHE tool suggests that validation of the conceptual model should involve face validity testing by sharing it with experts, and cross validity testing by comparing it with other conceptual models in the literature.

To test the face validity of the conceptual model, the model was shared with stakeholders (expert decision makers) who were asked if it was an accurate representation of reality, and whether the logic of the model made sense. Multiple stakeholders were involved in the development of the conceptual model (see Briggs et al. [1]) with nine of 12 stakeholders responding to an online survey saying that the model’s logic was plausible and had face validity. The PRIMEtime CE conceptual model structure was not shared with expert modellers, however the model is based on PRIMEtime, a previously published model which has been peer-reviewed and therefore critiqued by model experts [23].

The cross validity of PRIMEtime CE has been tested by comparing the model with similar multistate life table models in Australia and New Zealand upon which PRIMEtime was originally based [24–26]. Both PRIMEtime CE and the Australian and New Zealand models describe the same relationships between diet and disease, and physical activity and disease. Furthermore, PRIMEtime CE has the same basic model structure with the same primary disease outcomes as other models simulating the relationships between salt consumption, physical activity, and disease: changing salt consumption affects blood pressure and then cardiovascular disease outcomes [16, 19, 27–29], and changing physical activity affects IHD, stroke, diabetes, colorectal cancer, and breast cancer [14, 30–34].

#### Part B: input data validation

Input data validation assesses the appropriateness of data used to parameterise the model. The AdViSHE tool divides this into two parts, face validity testing of the input data and model fit testing.

The epidemiological data used to parameterise the model has been judged by experts as part of PRIME and PRIMEtime’s peer-review and found to be of sufficient quality for publication [23, 35]. Additional input data used by PRIMEtime CE are the costs, utilities, data relating to the interventions tested, and inputs parameterising the relationships between physical activity and disease. The costing methodology has been peer-reviewed and published elsewhere [12], as has the rest of the PRIMEtime CE model [1]. The utilities used in PRIMEtime CE are from a peer-reviewed journal and have also been peer-reviewed as part of other health economic models [11, 32, 36, 37].

Model fit testing asks whether the appropriate statistical tests have been performed where input parameters are based on regression models. Although many model input parameters used by PRIMEtime CE are based on regression models (such as utilities, disease relative risks, social care costs, and others), they are all from peer-reviewed secondary data sources (except for Wahid et al. parameterising the relationship between physical activity and different cancer subtypes [38]; see Briggs et al. for a full list of data sources [1]). Where primary data have been used, such as estimating NHS costs or how the interventions affect behavioural risk factors, no regression models were required.

#### Part C: validation of the computerised model

Validating the computerised model involves external review, extreme value testing, testing of traces, and unit testing.

The computerised model of PRIMEtime CE has been used by a separate team of researchers in the Nuffield Department of Population Health, University of Oxford, to build a related version of PRIMEtime CE with the BMI risk factor only in the programming language, R [39]. This has required understanding and reprogramming much of the model’s structure and its input data (as it relates to BMI), and during this exercise no major structural issues in the model were identified. Furthermore, the Burden of Disease Epidemiology, Equity & Cost-Effectiveness (BODE^3^) modelling team at the University of Otago, New Zealand [40] are in the process of evaluating the model as part of a model comparison exercise.

Extreme input values were modelled in PRIMEtime CE to see if outcomes were plausible and to detect any coding errors; results are shown in the Additional file 1: Table S7. Results for inputting either a 0% or 100% reduction in salt consumption as reported by NDNS food diaries are in line with what would be expected given the gains in QALYs and cost savings of the diet intervention results. Similarly, results with 100% of the population achieving recommended levels of physical activity or being sedentary are not implausible.

Testing of traces involves tracking individuals through the model by listing the number of people simulated and their disease status at various time points to ensure that the entire modelled cohort is accounted for. This is explicitly done in multistate life table models as the simulated population is listed in the life table, enabling the cohort and their associated disutility to be accounted for at all years.

Unit testing is the separate analyses of model submodules. When estimating the effect of an intervention on health, PRIMEtime CE does not have multiple submodules. There are two main parts to modelling the effect of an intervention on health and costs using PRIMEtime CE: the first is how the intervention affects a behavioural risk factor, and the second is how the change in behavioural risk factor then affects disease outcomes. The effect of the intervention on the behavioural risk factor will vary depending on the intervention being studied and the effect of changing the risk factor on health and costs is the basis of PRIMEtime CE and is discussed in detail in the cross-validation section below.

#### Part D: operational validation

This part of AdViSHE aims to validate model outcomes. Four different forms of operational validation are presented: face validity testing of model outcomes, cross validation testing, validation against outcomes using alternative input data, and validation against empirical data.

##### Face validity testing of model outcomes

To assess the face validity of outcomes, the modelled results should be shared with appropriate experts to see if the results make sense (both to decision makers and modellers). Model results were presented to health economists at the UK Department of Health and at Public Health England with verbal feedback suggesting that they are reasonable.

##### Cross validation and validation against outcomes using alterative input data

Cross validation of model outcomes involves comparing results with other models addressing similar problems to see what differences arise and to identify explanations for these differences. Validation against outcomes using alternative input data identifies how results might be affected with different data parameterising the model.

Cross validation testing of PRIMEtime CE outcomes was explored in detail by comparing results of the diet intervention with similar analyses published by the UK Health Forum using their microsimulation model [36], and by Collins et al. using the population level model, IMPACT CHD [16]. Results of the physical activity intervention were compared with results from Frew et al.’s bespoke population level Markov model [14]. To help explain differences in outcomes between Frew et al. and PRIMEtime CE, various input data from Frew et al. were used and results were compared. These three models were chosen for comparison because they either used a similar approach to estimating disease costs (the UK Health Forum model), or they provided some of the input parameters for the simulated interventions (IMPACT CHD and Frew et al.). PRIMEtime CE estimated similar disease outcomes to Frew et al. and slightly smaller effect sizes than Collins et al., but cost outcomes were significantly lower than those estimated by Collins et al. and higher than those estimated by Frew et al. When compared with the UK Health Forum model, differences in disease outcomes varied depending on the scenario simulated, but costs were much larger relative to the change in cumulative disease incidence. Details of the cross-validation can be found in pages 19 to 28 of the Additional file 1.

Estimates of the change in QALYs following the two modelled interventions were also compared with disease disability weights taken from the Global Burden of Disease study to calculate health adjusted life years (HALYs) [41]. Like QALYs estimated by PRIMEtime CE, HALYs are calculated by weighting the total number of years of life lived by the population by the level of ill-health after the intervention compared with the baseline. For QALYs, disease weighting uses utilities where perfect health has a score of one and death has a score of zero. In PRIMEtime CE, utilities are measured with the EQ-5D questionnaire and valued using time-trade off (the length of time in poor health someone would be willing to give up in order to live for a shorter amount of time in perfect health) [11]. Being in ill-health results in a utility decrement and a reduction in the average population utility. By contrast, disability weights value perfect health as zero, with ill health leading to additional disability on scale of zero to one. Disability weights are measured based on paired comparison questions where study participants are asked to choose which of two hypothetical individuals with different disease states they think is healthier [42]. To estimating HALYs in PRIMEtime CE, age and sex specific prevalent years lived with a disability were taken from the Global Burden of Disease study. These reflect the total population health loss associated with a disease, calculated using disease disability weights that are specific to different stages of disease and the prevalence of disease in the population across those stages. The average disability experienced by a given age and sex group is calculated by dividing the total age- and sex-specific prevalent years lived with a disability by the number of prevalent cases in that age and sex group. This value was subtracted from one and multiplied by the total number of years lived by the population with and without the intervention. This provides a comparison with the utility decrements used for estimating QALYs. Although measured very differently, comparisons between QALYs and HALYs are broadly similar (see Additional file 1: Table S11), providing additional evidence that the QALY estimates are valid.

##### Validation against empirical data

Validation against empirical data can take two forms, dependent (or internal) validation against data used to parameterise model, and independent (or external) validation against data not used by the model. For reasons of data availability, neither dependent nor independent validation of PRIMEtime CE has been completed. The reasons for this are outlined in the discussion.

#### Part E: other validation techniques

Part E of the AdViSHE tool suggests other types of validation that may also be used, such as guiding people through the conceptual model or computer model step by step (structured walk-throughs), or using naïve benchmarking against what might be expected from rough calculations. Health economists from the UK Department of Health and Public Health England have been guided through the conceptual model, and multiple colleagues in the Nuffield Department of Population Health, Oxford University, have been walked through the computer model, including facilitating the model being replicated in R. Other validation techniques have not been performed.

## Discussion

This paper uses PRIMEtime CE to estimate the cost effectiveness of reformulating salt and of expanding the Birmingham Be Active scheme across England. These results are then used with the AdViSHE tool to validate the PRIMEtime CE model. The modelled interventions illustrate how meeting the 2017 FSA salt reformulation target would be a more cost effective 10-year investment than expanding access to leisure centres across England. A key feature of PRIMEtime CE is that it adopts a standardised and replicable approach to identifying costs and utilities across multiple diseases related to diet and physical activity, allowing decision makers to directly compare unrelated interventions affecting these risk factors.

Using the AdViSHE tool provides evidence that the face validity of PRIMEtime CE’s conceptual model and its model inputs appear to be good, and the model also produces expected results when analyses are run using extreme input variables. Cross-validation efforts show that PRIMEtime CE does not systematically over or under estimate cost and disease outcomes compared with other models, but instead the magnitude and direction of the differences varies with each scenario and model comparison.

### Model validation

The absolute cost per QALY is heavily dependent on decisions made in three aspects of PRIMEtime CE: the choice of model scope and baseline settings, the choice of model parameters, and the choice of model structure.

Decisions about the scope - what is included in the model, for example what diseases or risk factors are chosen - and baseline settings such as the discount rate or time horizon can have significant implications for the final cost per QALY estimated. For example, sensitivity analyses using different time horizons resulted in the cost per QALY for salt reformulation being as high as £2.1 m (£0.9 m to £6.8 m) after one year, compared to being dominant after just nine years (Additional file 1: Table S6). Similarly, implementing Be Active across England had a cost per QALY of £12.2 m (£8.2 m to £18.5 m) after one year compared to £92,000 (£62,000 to £137,000) over the cohort’s lifetime.

The absolute cost per QALY also depends on the choice of model input parameters. Parameters used in PRIMEtime CE were chosen based on the aim of developing a cost-effectiveness model that can compare different interventions affecting multiple diseases. Although the uncertainty associated with each parameter used in PRIMEtime CE is quantified and represented by uncertainty intervals, cross-validation shows how the median cost per QALY and its uncertainty intervals change when different parameters are used. For example, we adapted PRIMEtime CE so that it included the same scope and baseline settings as Frew et al. (3.5% discount rate, a five-year time horizon, NHS costs only, and no unrelated disease costs) [14]. PRIMEtime CE then estimated the cost per QALY to be £1,711,000 (£1,252,000 to £2,400,000) compared with £400 estimated by Frew et al. However, changing the intervention costs, disease costs, and utility input parameters in PRIMEtime CE to match those used by Frew et al. reduced the cost per QALY estimate to £2700 (£2200 to £3300), see Additional file 1: Figure S11. This result is over 600 times smaller than the previous estimate and emphasises how different costs and utilities can dramatically affect results, and therefore subsequent policy decisions, despite parametric uncertainty being quantified and reported.

Finally, the model structure has an impact on the model’s final results. The model structure determines what input data are required and the relationships between the interventions, risk factors, and outcomes modelled. For example, as a multistate life table model, PRIMEtime CE includes age and sex specific data on baseline disease incidence, prevalence, case fatality, and trends, none of which are required by the Markov model used by Frew et al. [14] Both models also relate their input data to outcomes differently, for example, the prevalence of type two diabetes affects the risk of developing IHD and stroke in PRIMEtime CE, but not in Frew et al.’s model. Similar differences also exist between PRIMEtime CE and the UK Health Forum and Impact CHD models (see Additional file 1, pages 19 to 29) [16, 36]. These structural differences are likely to go some way to explain residual variations in model outcomes when model scope, baseline settings, and parameters have been standardised.

Differences between the cost-per-QALY estimates of PRIMEtime CE and the models used for cross-validation demonstrate the need for more work to be conducted in the area of structural uncertainty of public health models, following the lead of other disciplines such as the Agricultural Model Intercomparison and Improvement Project [43] and the Mount Hood Challenge comparing diabetes risk prediction models [4]. The development of reporting guidelines specific to public health modelling studies would also help when making model comparisons and allow model users to understand more clearly the impact of different model limitations (the principal limitations of PRIMEtime CE can be found in Briggs et al. [1]) [44].

The principal problem and challenge underlying decisions regarding model scope, parameters, and structure is that the true cost per QALY is not known, there is no gold standard against which results should be compared. Both dependent and independent validation checks would provide additional evidence of the model’s validity. However, dependent validation for models simulating public health behavioural interventions has the difficulty of isolating the disease burden attributable to a single behavioural risk factor from the population level dataset used to parameterise the model. Studies that attempt to quantify this attributable disease burden, for example how trends in salt consumption have affected CVD outcomes, generally use modelling themselves [45, 46]. Another approach is to compare model results with future disease trends using the same datasets that informed the model parameters. However, not only does this method encounter the same problem of quantifying the independent effect of a change in a behavioural risk factor on disease outcomes, but disease outcomes can take many years to manifest with the additional difficulties of separating out other secular epidemiological trends.

Independent validation of public health models is more challenging and relies on data from a similar intervention in a different setting, either following a trial or natural experiment. Such data are not available for the two interventions modelled in this paper – or, for that matter, for most population level public health interventions - with trials of interventions such as salt reformulation being difficult to design. In the absence of trial data, independently validating how the modelled change in risk factor affects health using natural experiments has the same difficulties as dependent validation. In reality, if the true costs and consequences of an intervention were known then there would be no need for the model.

### Comparisons with other studies

Previous reviews have found that public health interventions are often cost effective or cost saving [47–50]. Owen et al. reviewed 200 cost effectiveness estimates from NICE public health guidance and found that 89% had a cost per QALY of less than £30,000 [47]. Most analyses reviewed by Owen et al. used a lifetime horizon and PRIMEtime CE estimated that salt reformulation would be cost saving over the life time of the cohort. Furthermore, using PRIMEtime CE we estimate that expanding Be Active would have a lifetime cost per QALY of £92,000 (£62,000 to £137,000), not overly dissimilar to the free swimming for children and young people intervention included in the Owen et al. review which had a cost per QALY of £40,462. Masters et al. reviewed studies estimating the return on investment or cost benefit ratio of different public health interventions and found that the median return on investment among the 52 studies included was 14.3 to 1 [48]. PRIMEtime CE estimated that the lifetime return on investment of the salt intervention was £17 for every £1 spent, and would be higher if we included the economic benefit from health gains. Furthermore, the WHO Regional Office for Europe reviewed the evidence on the cost-effectiveness of public health interventions finding that many are cost-effective [49]. This review cited the WHO report on the “best buys” for the most cost-effective approaches to reducing the burden of NCDs in low- and middle-income countries [51]. The report suggests that in low- and middle-income countries, reducing salt consumption through reformulating unhealthy food and mass media campaigns would be very cost effective (the cost of an additional year of healthy life would be less than the national per person annual gross domestic product), as would promoting physical activity through mass media campaigns.

Results presented in this paper align with these reviews, however the intended use of PRIMEtime CE is not to add to the evidence base for individual interventions, but to allow the cost-effectiveness of different public health policies to be compared and ranked. Such rankings have been produced by the ACE Prevention programme of research in Australia [52], and the BODE^3^ programme in New Zealand [40]. The use of country specific data sources mean that results from the ACE and BODE^3^ programmes of research may not be directly applicable to England. Neither programme has investigated a scheme similar to Be Active, however both ACE and BODE^3^ simulated interventions aimed at reducing salt consumption finding that as with PRIMEtime CE, reformulation would dominant over a lifetime horizon [25, 53].

### Strengths and limitations

PRIMEtime CE has several strengths, it can directly compare interventions affecting different behavioural risk factors using the same input data and parameters, and the same assumptions and biases. There are important limitations of both data sources and the model’s structure, including the assumption that diseases are independent of one another. The choice of time horizon and model perspective will also impact results (as demonstrated in the sensitivity analyses). For example, using a short time-horizon will disproportionately impact younger people in the population as health benefits from preventive interventions have less time to accrue. The main strengths and limitations of PRIMEtime CE are discussed in detail elsewhere [1].

The modelled interventions have some important strengths and limitations. Both interventions use data representative of the English population to parameterise the distribution of the risk factor in the population, and both include wider costs to the government and industry where appropriate. However, both scenarios make assumptions about how the intervention is implemented and the sustainability of new behaviours. These strengths and limitations are expanded in detail in the Additional file 1.

### Future work

We hope to develop PRIMEtime CE to include more risk factors, such as smoking and alcohol consumption, and more disease outcomes as new data become available. In terms of model validation, newer large cohort studies such as the UK Biobank are providing datasets with enough information on both predictor variables and outcomes to potentially enable independent validation of public health models affecting behavioural risk factors [54]. For example, within the UK Biobank it might be possible to identify a subpopulation that consumes a steady level of sodium throughout their follow up, and another subpopulation whose salt consumption falls at some point post recruitment. The effect of this change in salt consumption could then be modelled with resulting change in health outcomes compared with the cohort’s subpopulations.

## Conclusions

In this paper we use PRIMEtime CE to estimate the cost-effectiveness of two different public health measures, salt reformulation and the expansion of the Be Active scheme. We then use the AdViSHE tool to help validate the model, highlighting the impact on results of model scope, input data sources, and model structure. We hope that future work can expand on these validation efforts, eventually validating model results against an independent data source.

## Additional file

Additional file 1: Additional methods for modelled interventions; additional results of the modelled interventions; strengths and limitations of the modelled interventions; cross validation of PRIMEtime CE; additional tables; and additional figures. (DOCX 763 kb)

## Data Availability

Datasets used and analysed in this study are publicly available and are either reported in the additional data file or are listed with appropriate references. This with the exception of draft Be Active programme evaluation data supplied to the corresponding author by Birmingham City Council, and specialised services commissioning expenditure data provided by NHS England, both of which the corresponding author does not have permission to share. These data can be made available with permission from the corresponding author as well as Birmingham City Council or NHS England respectively. All other data used or generated by the study are available from the corresponding author on request.

## Abbreviations

ACE: Assessing cost-effectiveness
BMI: Body mass index
BODE^3^: Burden of Disease Epidemiology, Equity & Cost-Effectiveness
COPD: Chronic obstructive pulmonary disease
CRA: Comparative risk assessment
FSA: Food Standards Agency
HTA: Health technology assessment
IHD: Ischaemic heart disease
ISPOR-SMDM: International Society for Pharmacoeconomics and Outcomes Research-Society for Medical Decision Making
NCDs: Non-communicable diseases
NDNS: National Diet and Nutrition Survey
NHS: National Health Service
NICE: National Institute for Health and Care Excellence
QALY: Quality adjusted life year
UI: uncertainty interval
WHO: World Health Organization
