# Supplementary material for: Estimating the cost-effectiveness of salt reformulation and increasing access to leisure centres in England, with PRIMEtime CE model validation using the AdViSHE tool
Source: BMC Health Serv Res. 2019 Jul 16;19:489. doi: 10.1186/s12913-019-4292-x (PMC6631881; doi:10.1186/s12913-019-4292-x)
Supplement: Supplementary file 1 — Additional methods for modelled interventions; additional results of the modelled interventions; strengths and limitations of the modelled interventions; cross validation of PRIMEtime CE; additional tables; and additional figures. (DOCX 763 kb) [file 12913_2019_4292_MOESM1_ESM.docx]

**Additional data file**

A D M Briggs, J Wolstenholme, P Scarborough. Estimating the cost-effectiveness of salt reformulation and increasing access to leisure centres in England, with PRIMEtime CE model validation using the AdViSHE tool.

**Contents**

**Additional methods for modelled interventions**………………….……………………………………..….2

**Additional results of the modelled interventions**……….…………………………………………………11

**Strengths and limitations of modelled interventions**……………………………………………………13

**Cross validation of PRIMEtime CE**………………………………………………………………………………….19

**Additional tables**……………………………………………………………………………………………………………30

**Additional figures**………………………………………………………………………………………………………….50

**References**………………………………………………..……………………………………………………………………61

**Additional methods for modelled interventions**

*Salt reformulation scenario modelling*

Calculating baseline salt consumption

The National Diet and Nutrition Survey (NDNS) is an annual cross-sectional survey of a representative sample of around 1,000 people in the UK. The survey asks individuals (or their parent/carer if under 12 years old) aged over 1.5 years from across the UK to complete four-day food diaries recording everything eaten or drunk, of which 4,156 diaries with at least three complete days were available for years 2008-2012 (3,441 diaries from people living in England). For individuals within the NDNS sample, data were available for approximately 11,000 different foods, aggregated into 61 food groups and 156 subsidiary food groups.

Individual level NDNS data for 3,441 individuals living in England who completed either three or four days of their food diary were used to calculate the average salt consumption by age group (0-3, 4-12, 13-17, 18-34, 35-54, and over 55 years) and by sex.[1] To do this, food diary data by age and sex were extracted and weighted within each age and sex group using the relevant NDNS weighting variable to adjust for non-response by adults and children, thereby ensuring the sample was representative of the UK population. The average salt content per 100g of the 156 subsidiary food groups was calculated separately for each age and sex group. This was then multiplied by the weight of each food group consumed at the individual level for each age and sex group to calculate the average daily salt consumption per person for all food recorded in the food diaries.

Meeting the Food Standards Agency 2017 ‘at home’ salt targets

Salt consumption following the intervention was calculated by changing the average salt content of each of the 156 subsidiary food groups to the ‘at home’ average 2017 salt target before again multiplying by the weight of each subsidiary food group consumed at the individual level for each age and sex group. The maximum salt target was used where no average target was specified, and no change to the salt content of a subsidiary food group was made following the intervention where the 2017 target was greater than the NDNS recorded salt content.

The method used to calculate salt consumption following the intervention assumes that nobody changes the food they eat following the intervention and that all out of home consumption recorded by NDNS food diaries achieves the ‘at home’ salt targets. For 19 of the NDNS subsidiary food groups, the included products were a composite of different foods included in the 2017 targets and in these circumstances the average salt target was used. It was not possible to weight the salt targets for these 19 food groups by the mass of each food-type consumed because this would require applying the relevant target to each of 11,000 individual foods included in NDNS, which was beyond the scope of this work. For the NDNS subsidiary food group, *1R other cereals*, couscous was not included under a salt target but all other foods were. In this case the salt target was applied to the entire category, assuming that couscous was a minor contributor to the category.

Estimating intervention costs

Industry costs are taken from Collins et al. who used salt reformulation costs of either £0 or £25,000 per product (£29,953 at 2014 prices).[2] The lower estimate is based on food products being reformulated as part of the natural product cycle and therefore absorbed within the food industry’s running costs, and the higher estimate is from the Food Standard Agency’s Impact Assessment 2009, which also suggests that up to 20,000 products would need to be reformulated.[3]

Government administrative costs were estimated using the WHO NCD costing tool, following methods used by Webb et al.[4, 5] The tool provides country specific resource unit costs (taken from the WHO-CHOICE database[6]) and it estimates the country specific inputs required for a range of national level interventions tackling tobacco consumption, alcohol misuse, poor diets, and physical inactivity. The amounts and types of resources required for each intervention vary across four phases of implementation: planning (year one), development (year two), partial implementation (years three to five), and full implementation (years six to 10). For PRIMEtime CE, the annual resources required for administering the salt intervention were based on programme management and meeting resources during the full implementation phase, as used by Webb et al. when they estimated the cost of the UK government’s sodium reduction strategy (see eTable 2 from Webb et al. supplemental materials for the full list of resources[5]). The administrative inputs used were multiplied by the UK specific unit costs from the WHO NCD costing tool, with costs inflated from 2008 to 2014 costs using the World Bank GDP deflator figures, and adjusted for England by scaling results by the ratio of the English population to the UK population (assuming that administrative costs are proportional to the size of the population).

There are no empirical estimates of uncertainty for industry or government costs and they are assumed to be “moderately uncertain” as defined by Blakely et al.[7] Therefore industry costs and government costs are separately allowed to vary in PRIMEtime CE according to a gamma distribution based on a normal distribution with a standard deviation of 10%.

Estimating the effect of salt consumption on disease

In PRIMEtime CE, salt consumption is estimated to affect IHD (ICD-10 I20-I25) and stroke (I60-I69) incidence via changing blood pressure. The parameter describing the relationship between salt and blood pressure is taken from a meta-analysis of randomised controlled trials and estimates a 5.8mmHg fall in systolic blood pressure for a 100mmol reduction 24hr urinary sodium excretion (equivalent to 5.9g salt [2,360mg sodium] consumption) after adjusting for age and baseline blood pressure.[8] For estimating the effect of a change in blood pressure on CVD incidence, age-specific parameters were taken from the prospective studies collaboration.[9] Uncertainty in all these parameters, as reported by the source articles, is included in PRIMEtime CE.[10]

Age and sex specific dietary data were extracted from NDNS using R x64 v3.2.0,[11] and were analysed using Microsoft Excel 2013.[12]

*Expanding the Be Active scheme and estimating the effect of physical activity on health*

Calculating baseline physical activity levels

The 2010-2011 Active People Survey (APS) provided data on the physical activity levels of 166,275 adults aged 16 years and over, after outliers reporting over 200 metabolic equivalent of task (MET) hours per week were removed.[13] A MET is defined as consuming 3.5ml of oxygen per kg of body weight per minute, the resting metabolic rate. Levels of physical exertion can be measured in relation to this resting metabolic rate, for example running at seven miles per hour is equivalent to 11 METs.[14] The APS from 2010-2011 was used because the raw data for this year were available and the computer code to calculate physical activity rates in MET hours/week for each individual was already written based on 2010-2011 APS variables (calculated by applying MET values provided by APS to the activities reported). Adults reporting zero MET hours/week were classed as sedentary. The adult population was divided into five-year age and sex categories and then into four physical activity categories: sedentary, under active, active, and recommended. Sedentary is equivalent to zero minutes of moderate physical activity per week, under active corresponds to more than zero but less than 60 minutes per week, active is equivalent to between 60 and 150 minutes per week, and recommended levels are based on the UK Chief Medical Officers’ Guidelines for adults of 150 minutes or more per week, assuming moderate physical activity corresponds to a minimum of three METs.[15]

Estimating the effect of Be Active on physical activity levels

Frew et al. measured the physical activity levels among a sample of individuals signing up to the Birmingham Be Active scheme over an eight-week period between 21 June, 2010 and 13 August, 2010.[16] All individuals enrolling during this time across 19 participating leisure centres (purposively selected to try and ensure that the participating sample was representative of the population) were invited to complete the Godin Leisure Time Exercise Questionnaire[17] at enrolment to measure pre-intervention physical activity levels. This was repeated three to four months later for post-intervention levels (this assumes that leisure centre attendance patterns are fixed by three months). In total, 2,163 (85% of participants) completed the baseline questionnaire, 1,156 (49%) completed the follow-up, and 797 (31%) completed both. The Godin Leisure Time Exercise Questionnaire asks how many times on average in the past week someone spent doing 30 minutes of either mild, moderate, or strenuous exercise, and provides examples of exercises that fall within each category. These data were then used to calculate the total number of MET minutes per week for each participant.

Physical activity levels for the 797 participants who completed both questionnaires were calculated by Frew et al. by assuming that strenuous activity is equivalent to 6-10 METs, moderate activity 3-5.9 METs, and mild activity <3 METs. From the resulting number of MET minutes per week, the participating population was categorised into three groups: inactive (labelled as under active in order to distinguish them from the sedentary population), active, and recommended; the participating population was assumed to be representative of the total population enrolled in the scheme.

A draft evaluation of the Be Active programme by the University of Birmingham, provided by Birmingham City Council (personal communication), reported the proportion of the population of Birmingham by age and sex registered in the scheme (taken from the Be Active database in 2010).[18] These data were used to estimate the proportion of the English population by five-year age and sex group that enrolled in the intervention. The ratio of men to women reported by Be Active was assumed to apply equally across all age groups. Using the 2014 English population, 3,764,078 adults were estimated to have enrolled in the modelled intervention.[19]

Estimating the effect of the Be Active scheme on the English population

The percentage of each age and sex group in each physical activity category in the English population following the intervention was calculated using data from Frew et al. Firstly, the percentage of each age and sex category that was classed as sedentary by APS was assumed not to change post-intervention. For the remaining active population, the percentage of those enrolled in the intervention that fell into each physical activity category pre- and post-intervention was calculated using data from Birmingham City Council[18], with the same percentages applied to each age and sex group. The percentage of the total active population in England in each physical activity category following the intervention was then calculated by adding the difference in the number of people in a given physical activity category pre- and post-intervention to the baseline number of people in that category. This was repeated for each age and sex group.

As an example, it was estimated from APS that 20% of males aged 50-54 (374,720 men) are sedentary, and that of the remaining active population, 55% (810,950) achieve recommended levels of physical activity. Using data from Frew et al., it was assumed that 8% (159,930) of this age and sex group enrolled in the intervention of which 60% (88,920) achieved recommended levels of activity at enrolment (pre-intervention), rising to 76% (112,880) following the intervention. Therefore, following the intervention, there were an additional 23,960 men aged 50-54 years achieving recommended levels of physical activity, and adding this to the baseline 810,950 men, the final number of men aged 50-54 years achieving recommended levels of physical activity was 834,910, or 45% of the age and sex group.

Estimating intervention costs

Intervention costs were estimated by Frew et al. to be £47 (£54 in 2014) per participant in year one and £26 (£30 in 2014) per participant in year two onwards, each was assumed to have a triangular distribution, as per Frew et al.[16] Intervention costs were calculated by dividing the total council spend on the scheme (including income replacement for the gyms, refurbishment costs, marketing, monitoring, and project management) by the number of participants.

Estimating the effect of physical activity on disease

The relationships between physical activity and IHD (ICD-10 I20-I25), stroke (I60-I69), type two diabetes (E11, E14), breast cancer (C50), and colorectal cancer (C18-C20) are included in PRIMEtime CE. The parameters describing the dose-response relationship between physical activity and disease and its uncertainty are taken from two meta-analyses of observational studies.[20, 21] Uncertainty in the size of the relationship between physical activity and disease (the beta-coefficient), and in the baseline physical activity levels are estimated from the input data sources and included in the overall model uncertainty estimates. More detail on the modelling of physical activity in PRIMEtime CE can be found elsewhere.[10]

Finally, uncertainty in the number participating in each age and sex group was estimated using the same approach used for salt reformulation industry and government costs and taken from Blakely et al. This assumes that the number participating is a “moderately uncertain” variable with a gamma distribution based on a normal distribution with a standard deviation of 10%.[7]

Age and sex specific population physical activity levels were analysed using STATA 14,[22] pre- and post-intervention physical activity distributions were calculated using Microsoft Excel 2013.[12]

**Additional results of the modelled interventions**

*Uncertainty analyses*

Parametric uncertainty was estimated using 2,000 iterations of a Monte Carlo simulation. This allows all model input parameters to simultaneously vary according to their probability distribution. The uncertainty interval for the cost per QALY for salt reformulation was

-£39,553 to £38,547, and £513,736 to £1,063,585 for the expansion of the Be Active scheme. The contribution of different parameters to this uncertainty is illustrated in the tornado plots in figures S6 and S7. The tornado plots show the size of the uncertainty interval when PRIMEtime CE only includes uncertainty resulting from either a single model input (such as the effect size of how a change in blood pressure alters stroke risk) or a collection of related model inputs (such as utility decrements). Model inputs leading to the greatest uncertainty are shown at the top of the plot.

*Sensitivity analyses*

Multiple deterministic sensitivity analyses were undertaken testing various assumptions in the model, a complete list of the sensitivity analyses is in table S1. The results of the sensitivity analyses are shown in table S6.

Salt reformulation

Changing the time horizon had a large influence on results for both interventions: the cost per QALY of reformulating salt was £2.10m (£0.97m to £6.78m) after one year, the intervention was dominant after nine years, and the return on investment was £16.50 (£5.70 to £33.30) for every £1 spent after 100 years (figure S8). Removing costs to industry also resulted in a significant increase in return on investment (£110.30 [£37.90 to £223.50] for every £1 spent) and reduced the cost per QALY. Removing government administrative costs made little difference to the cost per QALY but did increase the return on investment to £356.00 (£125.40 to £743.50) as annual intervention costs dropped from £826,125 to £255,233.

As a result of savings from social care being greater than NHS savings, the cost per QALY from an NHS perspective was £30,500 (£13,900 to £90,800) compared with being dominant with a return on investment of £1.20 (£0.40 to £2.50) from a social care perspective. However, in both cases the uncertainty intervals overlap with the result of the main reported analysis. Other sensitivity analyses had little effect on the overall cost per QALY compared with the main reported analysis.

Expanding Be Active

Changing the time-horizon significantly altered the cost-effectiveness of Be Active expansion, with results ranging from £12.18m (£8.20m to £18.47m) per QALY over a one year time horizon, to £91,700 (£63,000 to £137,400) per QALY if estimating outcomes over the lifetime of the cohort (figure S9). Other sensitivity analyses did not significantly alter the cost-effectiveness of the intervention, except for when modelling a reduction in physical activity over time which resulted in a cost per QALY of £1.05m (£0.76m to £1.52m). The reduction in physical activity assumed that 50% of those who moved from active to recommended levels of physical activity following the intervention, moved back to active levels after the first year.

**Strengths and limitations of modelled interventions**

*Salt reformulation*

The main strengths in the method used to estimate the impact of meeting the 2017 FSA targets on health are that it uses the NDNS four-year rolling programme (a representative sample of dietary habits in England), and that both government monitoring and administration costs of the intervention are estimated alongside industry costs.

There are limitations, however. The effect size of the intervention is based on salt consumption recorded in four-day food diaries reported between 2008 and 2012. These data suggest that average salt consumption among adults is 5.6g per day (6.4g among men and 4.9g among women). By contrast, the 2014 NDNS urinary sodium excretion survey reports that daily salt consumption among adults was 8.0g (9.1g salt consumed per day by men and 6.8g by women).[23] It is widely recognised that NDNS food diaries both under report total food consumption and also do not record salt added either at the table or during cooking.[24] If NDNS food diaries under report the amount of food (and therefore salt) consumed, then modelled results may underestimate the true effect on health and cost savings. The intervention assumes that the amount of salt added at the table or during cooking does not change, and the effect on health is estimated in PRIMEtime CE based on the absolute change in salt consumption rather than the relative change. In reality, the amount of salt added at home could change in either direction – reducing the amount of salt in food may lead people to add more at the table and during cooking to make up the difference, or conversely, as added salt in food declines, people’s palates may adjust meaning even less salt is subsequently added to food at home (as was the original premise of the UK salt reduction strategy[25]). Furthermore, the 2008-2012 food diary data used to estimate salt consumption by age and sex are assumed to apply to the 2014 population, assuming that salt consumption did not change between 2012 and 2014. This is likely to overestimate the effect of the intervention as it fails to account for declining trends in salt consumption.[23]

The intervention also assumes that at home salt targets are applied to all food consumed, including food eaten out. This is because it was not possible to distinguish between at home and out of home food consumption by subsidiary food group category, and NDNS data were not analysed at the individual food level. In 2014, it was estimated that 11% of total energy consumed was from eating out[26] and out of home food generally contains more salt (plus the out of home salt targets are higher) meaning that modelled results may over-estimate health benefits.

Regarding how the salt targets were applied to NDNS data, 19 of the 66 salt targets are maximum thresholds for a given food group rather than an average. Where only maximum thresholds were available, they were applied as an average salt target to the relevant NDNS subsidiary food groups meaning that final results may underestimate the reduction in salt consumption from these food groups. Similarly, for the 19 NDNS subsidiary groups where more than one salt target applied, an average of the salt targets was used which may either under- or over-estimate salt reduction in those categories.

Finally, the intervention does not account for any of the potential administrative and trade complexities surrounding food legislation. For example, costs to industry of reformulation may be passed on to the consumer in the form of price rises. This could lead to further reductions in salt consumption if people purchase fewer foods with higher prices, and to increases in purchases of other substituting foods. Similarly, it may be difficult to both measure and enforce salt reductions among imported foods, leading to a reduction in the possible effect size of the intervention and higher administration costs. Furthermore, compliance domestically may also not be perfect despite monitoring, thereby also reducing the intervention’s effect size.

*Expanding Be Active*

The strengths of using the described method to estimate the effect of expanding the Be Active scheme across England include using an empirical evaluation of the scheme to parameterise the change in physical activity levels and uptake rates by age and sex. Baseline data on physical activity levels in England were taken from the APS. This is the largest representative survey of adult physical activity in England meaning that physical activity levels can be estimated by five-year age group and by sex. The Health Survey for England also reports self-assessed physical activity levels however their sample size is smaller (8,291 adults included compared to 166,275 in APS) and the questionnaire used by APS is more representative of the physical activity data collected in the Be Active evaluation.[27] Other strengths of the method used include adjusting the relationships between physical activity and CVD to avoid over-estimating effects from diabetes (see Briggs et al.[10]), and quantifying the uncertainty associated with input parameters including baseline physical activity levels, the relationships between physical activity and disease, the number participating in the intervention, and intervention costs.

There are also some important methodological limitations. The comparator for the intervention is ‘no Be Active scheme’, therefore assuming that similar schemes are not in place elsewhere in England. If other schemes are in place, then the overall effect size may be overestimated. Furthermore, the intervention assumes that the scheme can be replicated elsewhere in England with the same age and sex specific uptake rates. Be Active is based in an urban environment with a high density of local authority leisure facilities. More rural settings without such well-established networks of leisure centres may not have the infrastructure to either implement the scheme or support similar uptake rates, meaning overall effect sizes may be overestimated. Similarly, the cost of the intervention to local authorities may vary by region and rather than using one intervention cost estimate from Frew et al.,[16] a bottom up costing exercise across multiple and varied English local authorities would likely provide a better estimate. Intervention costs were assumed to have a triangular distribution (also taken from Frew et al.) which means that our mean costs will be higher than the £53.80 cost per participant. Using a skewed distribution would reduce the mean cost to be closer to £53.80 and would reduce the total cost per QALY estimate, but would mean that results were less directly comparable to Frew et al. Reported Be Active uptake rates also varied by socio-economic group and by ethnicity.[18] At the time of the intervention, 19.8% of the population in Birmingham were from the most deprived quintile and 20.6% from the least deprived, however 23.2% in the most deprived quintile compared to 17.9% in the least deprived registered for the scheme. Therefore, Be Active has the potential to reduce socioeconomic inequalities. Uptake by ethnicity suggested that non-white individuals were more likely to participate than white. However, results of the intervention were not adjusted for socio-economic status or ethnicity because participation rates for these subgroups were not available by age or by sex. Also, uncertainty in the percentage of individuals in each physical activity category pre- or post-intervention was not explicitly included in the model. This uncertainty is in part captured by allowing the mean physical activity level in each category to vary for each age and sex group according to its standard error. However, overall uncertainty in the model outcome may be underestimated.

Physical activity levels recorded in both the APS and the Be Active evaluation are self-reported and may not accurately represent pre- and post-intervention physical activity rates. Given that the direction of bias for the self-reported physical activity levels is unknown, reported results may under- or over-estimate the true effect of the intervention.[28] The intervention also assumes that there is no change in the sedentary population following the intervention. If some of those engaging in the intervention are sedentary at baseline (classified as inactive by Frew et al.) then the effect size of the intervention may be under-estimated. Furthermore, the Be Active evaluation assumes that post-intervention physical activity rates do not change from three months post intervention, however this may overestimate the true effect size if, as with some other physical activity interventions, the effect size attenuates beyond three months post intervention.[29] This is explored in a sensitivity analysis replicating one of Frew et al.’s sensitivity analyses: half of those who move from active to recommended levels of physical activity after the intervention return to the active category after one year.[16]

Furthermore, as described in Briggs et al.[10], Wahid et al. included additional physical activity domains that are not included in the Be Active evaluation or the APS.[13, 16, 20, 21] This may over-estimate the health benefit because these are larger at lower levels of physical activity. Baseline physical activity data was taken from the 2010-2011 APS. According to the APS, rates of participation in active recreation three or more times a week have increased from 21.8% in 2010/11 to 23.8% in 2015/16, although any sports participation has decreased from 48.5% to 46.6% over the same period. Data on physical activity rates are only available from 2012 to 2014 and over these three years, the percentage of people inactive decreased from 29.4% to 27.7% and the percentage active increase from 54.9% to 57.0%.[30] If physical activity levels did increase between 2010/11 and 2014 (the start of the intervention), then the intervention may again over-estimate health benefits because there are greater health gains for increases in physical activity levels from a lower baseline.

Finally, PRIMEtime CE only models the relationships between physical activity and IHD, stroke, type two diabetes, colorectal cancer, and stroke. Other studies have modelled relationships with other diseases including dementia[31] and depression,[32] and there is increasing evidence of physical activity reducing the risk of other cancer subtypes.[33] Therefore, the overall health benefits of the intervention may be underestimated.

**Cross validation of PRIMEtime CE**

*Cross validation of the salt reformulation results*

The UK Health Forum estimated the effect on CVD burden and NHS costs of four different salt consumption scenarios in England over a period of 20 years from 2014 to 2034:

1. following current trends and reaching 8.0g salt per day consumed by men and 6.1g per day consumed by women in 2034;
2. a best case where mean salt consumption for both men and women reaches 5.0g per day in 2034;
3. a worst case where salt consumption stays at 2014 levels of 8.9g per day for men, and 6.8g per day for women;
4. an expected scenario where mean consumption reaches 7.0g per day for men and women in 2034.[34]

In order to estimate the effect on health of the UK Health Forum scenarios using PRIMEtime CE, PRIMEtime CE was adapted to include many of the UK Health Forum microsimulation model baseline settings. For each of the scenarios, 2014 age and sex specific salt consumption in PRIMEtime CE was adjusted such that the average baseline consumption was 8.9g per day for men and 6.8g per day for women to match that in the UK Health Forum scenario. Salt consumption was then assumed to change with a linear trend over 20 years towards the 2034 target, with the sex specific salt consumption target in each of the four scenarios being the same across all age groups. A discount rate of 0% was used and the time horizon was set to 20 years with only those over the age of 18 years included. As with PRIMEtime CE, the UK Health Forum model uses a top down method to estimate the cost per prevalent case to the NHS with disaggregated NHS England programme budgeting data. The 2013/14 disease costs used in PRIMEtime CE were replaced by the 2012/13 disease cost estimates for MI and stroke used by the UK Health Forum; societal costs, intervention costs, and costs from other unrelated diseases were removed.

Other differences in input data between the two models remained. The UK Health Forum estimated the effect on MI rather than IHD, but the impact of this on model outcomes is likely to be minimal as costs are taken from the CHD NHS England programme budgeting category (the same as used in PRIMEtime CE), and the relative risk of CHD following a change in blood pressure was also the same in both models.[9] Baseline disease incidence and prevalence data differed between the two models. The average baseline prevalence rates for MI and stroke used by the UK Health Forum were 1,474 per 100,000 and 1,188 per 100,000 compared to 1,819 per 100,000 and 2,141 per 100,000 for IHD and stroke in PRIMEtime CE. Baseline incidence rates were also higher in PRIMEtime CE at 143 per 100,000 and 178 per 100,000 compared to 131 per 100,000 and 108 per 100,000 used by the UK Health Forum. The UK Health Forum used routinely reported British Heart Foundation data from between 2006 and 2010[35, 36] for incidence, prevalence, and case fatality rates (based on various sources including the Health Survey for England and the General Practice Research Database, GPRD). By contrast, PRIMEtime CE uses different data sources including primary research studies and DISMOD II.[10] Larger baseline prevalence and incidence rates in PRIMEtime CE would mean that the model is likely to estimate larger absolute changes in disease cumulative incidence than the UK Health Forum.

Estimates of baseline salt consumption by age and sex differed between the models. Both models used NDNS data with the UK Health Forum model estimating trends for each sex and for three age categories for men (20-39 years, 40-59 years, over 60 years) and two for women (20-39 years and over 40 years) based on urinary sodium excretion studies from between 2001 and 2011.[37] The proportion of the population in each age and sex group consuming less than 6g, between 6g and 12g and more than 12g salt per day was estimated for 2014 and each subsequent year by fitting a lognormal distribution to the data, with trends in the proportion of the population in each consumption category for each age and sex group estimated through to 2034. In PRIMEtime CE, age and sex specific mean salt consumption was estimated using NDNS dietary data from 2008 to 2012[1] across six age groups (0-3 years, 4-12 years, 13-17 years, 18-34 years, 35-54 years, and over 55 years; only those aged 18 years and older were included in the comparison with the UK Health Forum) and the mean was then scaled to match urinary sodium excretion with uncertainty estimated using standard errors from NDNS data. The UK Health Forum did not report their baseline salt consumption by age and sex, only the average for each gender, so it is difficult to know the direction in which these differences will affect results. For the purposes of the comparison, the change in salt consumption over time was modelled in the same way as the UK Health Forum.

The other important remaining difference between the two models was how the change in blood pressure was estimated following a change in salt consumption. Both models use He et al.,[8] with PRIMEtime CE using He et al.’s estimate that systolic blood pressures falls by an average 5.8mmHg following a 100mmol reduction in 24 hour urinary sodium (adjusted for age and baseline blood pressure, equivalent to 5.9g salt consumption). The UK Health Forum, by contrast, used results from a subgroup analysis suggesting that blood pressure among hypertensive adults falls by 5.4mmHg following a 75mmol reduction in 24-hour urinary sodium (7.2mmHg for a 100mmol reduction) and by 2.4mmHg (3.2mmHg) among normotensive adults. Furthermore, based on data from both He et al.[8] and Cappuccio et al.[38] suggesting that older adults have a greater fall in blood pressure for the same reduction in 24 hour urinary sodium excretion, the UK Health Forum model assumed that the effect size for hypertensive adults from He et al. applies to anybody over the age of 65 years. As those over 65 years have a greater burden of CVD, these differences mean that the UK Health Forum model is likely to estimate larger overall effect sizes than PRIMEtime CE.

The results of the UK Health Forum model alongside the modelling of equivalent scenarios in PRIMEtime CE are shown in table S8 and figure S10. The UK Health Forum estimated larger increases in stroke and IHD cumulative incidence than PRIMEtime CE when comparing the following trends scenario (salt consumption falling from 8.9g to 8.0g among men and 6.8g to 6.1g among women) with the worst case scenario (no change in salt consumption). This is likely due to the differences outlined above in how the relationship between salt consumption and blood pressure is parameterised. By contrast, changes in disease incidence measured by the UK Health Forum model and PRIMEtime CE were similar for the comparison of the best case scenario (salt consumption falls to 5.0g among both men and women) with the following trends scenario. In PRIMEtime CE, the 3.0g and 1.1g reductions in salt consumption among men and women respectively produced changes to cumulative incidence over the 20 years of the intervention that were much greater in magnitude than those estimated by the 0.9g and 0.7g increases in salt consumption simulated in the worst case scenario versus follows trends scenario. However, the UK Health Forum estimated that the reduction in disease cumulative incidence arising following the best case versus follows trends scenario was similar in magnitude to the increase estimated in the worst case versus follows trends scenario. This may also be due to differences in how the relationship between salt consumption and blood pressure is parameterised. As salt consumption declines to increasingly low levels, reductions in blood pressure would mean that fewer individuals in the UK Health Forum model are hypertensive and therefore those under 65 years old would have a smaller reduction in their risk of developing CVD compared to the population simulated in PRIMEtime CE where the risk reduction is the same irrespective of hypertension status. This means that at lower levels of salt consumption, PRIMEtime CE may be less likely than at higher levels of salt consumption to underestimate the health benefit when compared with the UK Health Forum model.

The UK Health Forum model also predicted larger changes to IHD incidence than stroke incidence for both the worst case versus follows trends and best case versus follows trends scenarios, with the pattern being the reverse with PRIMEtime CE. This reflects the differences in baseline disease incidence and prevalence rates between the two models.

Compared with the UK Health Forum model, PRIMEtime CE estimated the change to NHS costs to be proportionately larger for the number of cases averted. Healthcare costs were estimated in both models based on the annual prevalence of disease and therefore cases of MI and stroke that are averted soon after the intervention accrue more years’ worth of cost savings than those that are averted later. In PRIMEtime CE, the fall in salt consumption occurred evenly over the 20 years of the intervention with the same relationship between salt consumption and blood pressure irrespective of age and hypertension status. In the UK Health Forum model, the effect of reducing salt consumption on blood pressure was smaller than in PRIMEtime CE for normotensive individuals aged under 65 years, but higher for those aged over 65 years or with hypertension. As more of the 20 years were simulated, an increasing proportion of the modelled population would have either turned 65 years old or developed hypertension. Therefore, this would accelerate the decline in incidence rate towards the end of the 20 years with fewer years in which to accrue savings.

Finally, PRIMEtime CE results suggest that the ‘expected’ scenario would lead to small health gains when compared to following trends (although uncertainty intervals do overlap with zero), this is in contrast to the UK Health Forum model, which predicted small health losses. This is because the health gained by men moving from 8g salt consumption a day to 7g outweighs the health loss from women moving from 6.1g to 7g in PRIMEtime CE, whereas the balance of outcomes by sex is the other way around in the UK Health Forum model.

Collins et al. also estimated the effect on health and NHS costs of reformulating food to contain less salt, this time using the IMPACT CHD model.[2] The IMPACT CHD model is a population level model that estimates CHD mortality rates over time based on historical trends and the attributable disease burden of risk factors such as salt consumption. The authors estimated the effect on NHS costs and life years gained in England between 2010 and 2020 of 20%, 15%, and 2% reductions in salt consumption. Consumption was assumed to fall at the beginning of the 10 year period with health gains quantified for seven separate patient groups – acute MI admissions, unstable angina admissions, secondary prevention after acute MI, secondary prevention after revascularisation, angina in the community, heart failure admissions, and heart failure in the community. To enable comparisons of results, PRIMEtime CE was adapted to simulate the same reductions in salt consumption estimated by Collins et al., stroke was removed from the model so that only the effect on IHD was calculated, no unrelated disease costs were included, and the same 3.5% discount rate was used.

There remained some important differences between the two models, such as each model including different diseases. PRIMEtime CE models IHD, whereas IMPACT CHD simulated the effects of the intervention on a wider range of coronary heart diseases. Furthermore, the data sources for baseline disease incidence and prevalence differed between the two groups, with IMPACT CHD using pre-2011 data from Hospital Episode Statistics (HES),[39] the Myocardial Ischaemia National Audit Project,[40] and GPRD,[41] compared to the data sources used by PRIMEtime CE (see Briggs et al.[10]). Collins et al. did not publish their baseline disease incidence and prevalence rates making it unclear how this would affect results. Both models used He et al. to estimate the effect of a change to salt consumption on blood pressure.[8] However, the IMPACT CHD model uses the separate estimates for adults with and without hypertension to quantify an average effect size for the population in each of the seven patient groups. Again, the effect of this difference on results is not clear because the final effect sizes used by Collins et al. are not reported.

A further difference between the two models is that although both use Lewington et al. to estimate how a change in blood pressure affects vascular disease,[9] this is only used for mortality estimates by IMPACT CHD to quantify life years gained, compared to incidence in PRIMEtime CE. In order to estimate the number of life years gained in IMPACT CHD, the number of deaths delayed or postponed following the 10 years of the intervention was quantified based on the change in blood pressure and multiplied by the age specific median-survival for the those with diagnosed CHD, undiagnosed CHD, and without CHD. This therefore assumes that the same survival benefit occurs, irrespective of from when the death is postponed following the intervention. In PRIMEtime CE, the life years gained was estimated based on the difference in 10 year life expectancy for each age group calculated as the difference in the sum of person-years lived pre- and post-intervention. This method does not use an estimate of median-life expectancy and is likely to underestimate the number of life years gained compared to IMPACT CHD because rather than assuming all deaths are delayed from the beginning of the intervention, deaths are delayed across the ten years.

Finally, there are significant differences in cost estimates. In contrast to PRIMEtime CE, IMPACT CHD uses bottom-up estimates from reference costs based on expected treatments, medications, and professional health visits for each patient group simulated. For example, a patient with heart failure in the community has annual costs based on using four different medications and having six GP and six practice nurse appointments a year. Costs are saved as a result of fewer individuals developing disease, based on the attributable fraction of CHD cases for the change in blood pressure using odds ratios from the INTERHEART study.[42] Neither the number of cases averted nor the unit costs for each patient group are reported by Collins et al. making it difficult to understand how these differences impact on the final NHS cost savings. Results of the interventions modelled by Collins et al. using IMPACT CHD and PRIMEtime CE are shown in table S9.

The estimated number of life years gained are smaller using PRIMEtime CE than IMPACT CHD, however they are in the same order of magnitude. It is not possible to know how much of the variation is due to differences in the diseases simulated and the calculation of life years gained, and how much is due to other aspects of each model’s structure. The changes to cost estimates are markedly different with NHS savings using PRIMEtime CE being roughly proportional to the number of lives saved. By contrast, significantly higher cost savings are estimated using IMPACT CHD, with only a 37% difference in savings for a 2% reduction in salt consumption compared to a 20% reduction, despite a 90% difference in life years gained. This implies that the IMPACT CHD cost savings are more aligned with changes in morbidity than mortality, about which there is not enough detail in the paper and its supplementary materials to draw conclusions as to the main reasons for differences in outcomes between the two models.

*Cross validation testing of physical activity results*

PRIMEtime CE was compared with Frew et al.’s Markov model, which estimated the cost-effectiveness of the Be Active intervention.[16] The input parameters quantifying the effect of Be Active on physical activity used by PRIMEtime CE were taken from Frew et al. and when the cost-effectiveness of scaling up the intervention to England was estimated, the cost per QALY was £727,000 (95% UI £514,000 to £1,064,000), see table 2 in the main paper, compared to £400 per QALY estimated by Frew et al. The PRIMEtime CE results are calculated using a different time horizon, cost perspective, and discount rate than Frew et al. When using 3.5% discounting, a five-year time horizon, and NHS costs only with no unrelated disease costs, as used by Frew et al., the cost per QALY was even higher at £1,711,000 (£1,252,000 to £2,400,000).

However, the two models have important differences between their input parameters, in particular the disease specific utility and cost estimates. Frew et al. use a variety of sources for their utility decrements and costs, with both being generally larger than those used in PRIMEtime CE. For example, the utility for stroke used by Frew et al. is 0.612 (from a baseline of 0.805, 0.875, or 0.912 depending on the baseline state of physical activity) compared with a utility decrement of -0.112 (incident case) and -0.073 (prevalent case) used by PRIMEtime CE. Furthermore, the cost per incident and prevalent stroke case is £9,630 and £2,396 respectively in Frew et al.’s model, compared with £843 for both incident and prevalent cases in PRIMEtime CE.

The other important difference between the two models is how they quantify the risk of disease following a change in physical activity levels. Frew et al. conducted their own systematic reviews and meta-analyses focusing on leisure time physical activity – as is measured in the Be Active evaluation - not including occupational or domestic physical activity. They do not report a dose-response relationship, meaning that these cannot be used to estimate the effect of the intervention on different age and sex groups where the baseline physical activity levels differ. By contrast, PRIMEtime CE uses results of two meta-analyses providing a dose response relationship of total physical activity and disease outcome.[20, 21] Table S10 compares the relative risks used by both studies, with those used by Frew et al. for people moving between under active to recommended levels of physical activity being significantly smaller than those used by PRIMEtime CE. Furthermore, the annual disease risks used by Frew at al. are significantly greater than the baseline disease incidence rates in PRIMEtime CE. For example, the annual risk of breast cancer used by Frew et al. was 0.021, 0.018, and 0.017 depending on whether an individual was under active, active, or achieving recommend physical activity levels. By contrast, the average baseline annual incidence rate of breast cancer among women aged 16 years and over used in PRIMEtime CE was 0.0021 (varying by age). Therefore Frew et al.’s model is likely to lead to both greater relative and absolute health gains than PRIMEtime CE.

To compare PRIMEtime CE with Frew et al.’s model, the costs and utilities used by PRIMEtime CE were replaced with those used by Frew et al. Furthermore, PRIMEtime CE was adapted to include 2010 intervention costs, a five-year time horizon, an NHS perspective, and a 3.5% discount rate. The resulting cost per QALY was £2,662 (£2,176 to £3,327), much closer to Frew et al.’s estimate of £400 per QALY. Figure S11 shows the results of the PRIMEtime CE comparisons with Frew et al. on the cost effectiveness plane. The residual difference in cost per QALY between the two models was due to costs: the incremental QALY estimate was 0.06 per person in Frew et al. and 0.06 (0.05 to 0.07) in PRIMEtime CE, but the incremental cost was £24 in Frew et al. compared to £153 (£116 to £206) in PRIMEtime CE. This cost difference may in part be due to how disease relative risk estimates vary between the two models as differences in individual disease outcomes may have led to a similar overall effect on QALYs but not on costs, and in part be due to different underlying model structure.

**Additional tables**

Table S1. List of sensitivity analyses. Updated from Briggs et al.[10]

| **Sensitivity analysis** | **Explanation of what is changed compared to the initial analysis** |
| --- | --- |
| Changing the time horizon. | Time horizon changing from 10 years in the main analysis to one year, five years, 20 years, and 100 years (lifetime of the cohort). |
| Analysing results from an NHS perspective. | Estimating cost effectiveness using the change in NHS costs and intervention costs only (without any societal costs). |
| Analysing results from a social care perspective. | Estimating cost effectiveness using the change in social care and intervention costs only (without any NHS costs). |
| Including social care costs and productivity. | Adding an economic estimate of changes to productivity arising from the intervention. |
| Including all wider societal costs. | Including an economic estimate of the intervention on all wider societal costs. |
| Using a discount rate of 3.5%. | Changing the discount rate for costs and outcomes from 1.5% to 3.5%. |
| No disease costs estimated for diseases not explicitly modelled by PRIMEtime CE (unrelated disease costs). | Removing from the model any NHS and social care costs estimated to accrue due to diseases that are not explicitly modelled by PRIMEtime CE. |
| No cancer included in the model | Cancer removed from the model so that only IHD, stroke, type two diabetes, and liver cirrhosis are included. |
| Only including diseases directly related to the risk factor affected. | For the diet intervention, only IHD and stroke are included in the model, and for the physical activity intervention, IHD, stroke, type two diabetes, breast cancer, and colorectal cancer are included. |
| No delay in the introduction of the intervention (salt reformulation only). | The effects and costs of the diet intervention come into full effect immediately rather than over a three-year period. |
| No industry costs included (salt reformulation only). | The cost to industry is not included in the intervention’s costs. |
| Not including industry costs or government administrative costs (salt reformulation only). | Only included costs from government monitoring. |
| Fall in physical activity over time (Be Active expansion only). | Physical activity levels are not sustained following the intervention with 50% of individuals who move from active to recommended moving back to active one year after the intervention. |

Table S2. Sodium and salt consumption before and after the salt reformulation intervention

| **Age and sex group** | **Baseline** (SE) | | **Intervention** (SE) | | **Difference** (SE) | |
| --- | --- | --- | --- | --- | --- | --- |
|  | Sodium (mg) | Salt (g) | Sodium (mg) | Salt (g) | Sodium (mg) | Salt (g) |
| 0-3 male | 1,314 (70) | 3.3 (0.2) | 1,078 (55) | 2.7 (0.1) | 237 (0.5) | 0.6 (0.04) |
| 4-12 male | 1,982 (55) | 5.0 (0.1) | 1,596 (43) | 4.0 (0.1) | 387 (0.6) | 1.0 (0.03) |
| 13-17 male | 2,604 (109) | 6.5 (0.3) | 2,078 (86) | 5.2 (0.2) | 526 (0.9) | 1.3 (0.06) |
| 18-34 male | 2,768 (145) | 6.9 (0.4) | 2,201 (115) | 5.5 (0.3) | 566 (1.1) | 1.4 (0.08) |
| 35-54 male | 2,528 (79) | 6.3 (0.2) | 2,073 (63) | 5.2 (0.2) | 455 (0.8) | 1.1 (0.04) |
| 55+ male | 2,354 (74) | 5.9 (0.2) | 1,931 (60) | 4.8 (0.1) | 423 (0.7) | 1.1 (0.04) |
| 0-3 female | 1,271 (73) | 3.2(0.2) | 1,047 (58) | 2.6 (0.1) | 224 (0.5) | 0.6 (0.04) |
| 4-12 female | 1,790 (48) | 4.5 (0.1) | 1,446 (38) | 3.6 (0.1) | 343 (0.5) | 0.9 (0.03) |
| 13-17 female | 1,931 (74) | 4.8(0.2) | 1,551 (58) | 3.9 (0.1) | 380 (0.7) | 0.9 (0.04) |
| 18-34 female | 2,076 (93) | 5.2 (0.2) | 1,663 (74) | 4.2 (0.2) | 413 (0.8) | 1.0 (0.05) |
| 35-54 female | 1,938 (50) | 4.8 (0.1) | 1,615 (41) | 4.0 (0.1) | 323 (0.5) | 0.8 (0.03) |
| 55+ female | 1,869 (58) | 4.7 (0.1) | 1,556 (47) | 3.9 (0.1) | 313 (0.5) | 0.8 (0.03) |

SE, standard error

Table S3. Change in modelled disease incidence by sex after the salt reformulation intervention

|  | **Males** | **Females** | **Total** |
| --- | --- | --- | --- |
| Change in IHD cumulative incidence [incidence rate per 100,000] | -11,637 [-53.3]  (-18,375 to -5,027) | -4,927 [-21.6]  (-7,819 to -2,110) | -16,568 [-37.1]  (-26,223 to -7,167) |
| Change in stroke cumulative incidence [incidence rate per 100,000] | -13,840 [-63.4]  (-21,855 to 5,964) | -10,606 [-46.5]  (-16,666 to -4,545) | -24,460 [-54.8]  (-38,686 to -10,573) |
| Change in type two diabetes cumulative incidence [incidence rate per 100,000] | 54 [0.2]  (24 to 86) | 47 [0.2]  (20 to 73) | 101 [0.2]  (44 to 158) |
| Change in breast cancer cumulative incidence [incidence rate per 100,000] | 0 [0] | 16 [0.1]  (7 to 24) | 16 [0.0]  (7 to 24) |
| Change in colorectal cancer cumulative incidence [incidence rate per 100,000] | 20 [0.1]  (8 to 31) | 10 [0.0]  (4 to 16) | 30 [0.1]  (13 to 47) |
| Change in lung cancer cumulative incidence [incidence rate per 100,000] | 22 [0.1]  (9 to 34) | 11 [0.0]  (4 to 17) | 32 [0.1]  (14 to 50) |
| Change in stomach cancer cumulative incidence [incidence rate per 100,000] | 4 [0.0]  (2 to 6) | 2 [0.0]  (1 to 3) | 6 [0.0]  (2 to 9) |
| Change in pancreas cancer cumulative incidence [incidence rate per 100,000] | 3 [0.0]  (1 to 5) | 3 [0.0]  (1 to 4) | 6 [0.0]  (3 to 10) |
| Change in kidney cancer cumulative incidence [incidence rate per 100,000] | 4 [0.0]  (2 to 5) | 2 [0.0]  (1 to 2) | 5 [0.0]  (2 to 8) |
| Change in liver cancer cumulative incidence [incidence rate per 100,000] | 2 [0.0]  (1 to 3) | 1 [0.0]  (0 to 2) | 3 [0.0]  (1 to 5) |
| Change in liver cirrhosis cumulative incidence [incidence rate per 100,000] | 3 [0.0]  (1 to 5) | 2 [0.0]  (1 to 3) | 5 [0.0]  (2 to 7) |

Results presented are the median values from 2,000 iterations of a Monte Carlo simulation, as such, the numbers in the final column may not equal the sum of males and females; 95% uncertainty intervals in parentheses; IHD, ischaemic heart disease

Table S4. Percentage of the English population with different levels of physical activity by age and sex before and after the Be Active expansion

| **Age and sex group** | **Pre-intervention** | | | | | **Post-intervention** | | | | |
| --- | --- | --- | --- | --- | --- | --- | --- | --- | --- | --- |
|  | Sedentary | Under active | Active | Recommended | Sedentary | | Under active | Active | Recommended |  |
|  | 0 MET hrs/wk | <3MET hrs/wk | 3-7.5 MET hrs/wk | >7.5 MET hrs/wk | 0 MET hrs/wk | | <3MET hrs/wk | 3-7.5 MET hrs/wk | >7.5 MET hrs/wk |  |
| M16-19 | 6.2% | 9.0% | 9.9% | 74.9% | 6.2% | | 7.9% | 9.1% | 76.8% |  |
| M20-24 | 9.9% | 10.7% | 10.7% | 68.7% | 9.9% | | 9.6% | 9.9% | 70.6% |  |
| M25-29 | 12.0% | 13.9% | 11.5% | 62.6% | 12.0% | | 12.7% | 10.8% | 64.6% |  |
| M30-34: | 12.7% | 15.7% | 13.5% | 58.1% | 12.7% | | 14.5% | 12.8% | 60.0% |  |
| M35-39 | 14.4% | 17.6% | 14.9% | 53.1% | 14.4% | | 16.6% | 14.1% | 54.9% |  |
| M40-44 | 15.2% | 18.5% | 15.2% | 51.1% | 15.2% | | 17.4% | 14.4% | 53.0% |  |
| M45-49 | 16.8% | 19.3% | 14.8% | 49.1% | 16.8% | | 18.5% | 14.3% | 50.4% |  |
| M50-54 | 20.4% | 20.3% | 15.3% | 44.1% | 20.4% | | 19.5% | 14.7% | 45.4% |  |
| M55-59 | 24.4% | 21.1% | 14.0% | 40.5% | 24.4% | | 20.5% | 13.6% | 41.5% |  |
| M60-64 | 26.4% | 20.6% | 13.1% | 39.8% | 26.4% | | 20.0% | 12.7% | 40.9% |  |
| M65-69 | 27.9% | 18.8% | 14.0% | 39.3% | 27.9% | | 18.5% | 13.9% | 39.7% |  |
| M70-74 | 32.4% | 18.0% | 12.6% | 37.1% | 32.4% | | 17.7% | 12.4% | 37.5% |  |
| M75-79 | 40.9% | 16.0% | 10.9% | 32.2% | 40.9% | | 15.7% | 10.8% | 32.6% |  |
| M80-84 | 51.7% | 14.4% | 9.6% | 24.3% | 51.7% | | 14.1% | 9.5% | 24.7% |  |
| M85+ | 48.6% | 13.8% | 9.9% | 27.7% | 48.6% | | 13.5% | 9.8% | 28.1% |  |
| Overall | 23.0% | 17.4% | 13.2% | 46.4% | 23.0% | | 16.6% | 12.6% | 47.7% |  |
|  |  |  |  |  |  | |  |  |  |  |
| F16-19 | 13.8% | 17.4% | 16.0% | 52.8% | 13.8% | | 16.2% | 15.1% | 54.9% |  |
| F20-24 | 16.9% | 16.9% | 16.7% | 49.5% | 16.9% | | 15.7% | 15.8% | 51.6% |  |
| F25-29 | 14.4% | 19.5% | 17.9% | 48.2% | 14.4% | | 18.3% | 17.1% | 50.2% |  |
| F30-34 | 14.0% | 19.8% | 17.9% | 48.3% | 14.0% | | 18.6% | 17.0% | 50.3% |  |
| F35-39 | 14.7% | 21.6% | 18.0% | 45.7% | 14.7% | | 20.5% | 17.3% | 47.6% |  |
| F40-44 | 14.7% | 20.9% | 17.8% | 46.6% | 14.7% | | 19.7% | 17.1% | 48.5% |  |
| F45-49 | 16.9% | 20.5% | 16.3% | 46.3% | 16.9% | | 19.7% | 15.8% | 47.6% |  |
| F50-54 | 19.9% | 20.7% | 17.2% | 42.2% | 19.9% | | 19.9% | 16.7% | 43.5% |  |
| F55-59 | 21.8% | 22.0% | 17.8% | 38.4% | 21.8% | | 21.4% | 17.4% | 39.4% |  |
| F60-64 | 24.1% | 21.1% | 18.1% | 36.7% | 24.1% | | 20.4% | 17.7% | 37.8% |  |
| F65-69 | 28.2% | 20.5% | 16.4% | 34.9% | 28.2% | | 20.3% | 16.2% | 35.3% |  |
| F70-74 | 36.3% | 19.3% | 15.2% | 29.1% | 36.3% | | 19.1% | 15.1% | 29.5% |  |
| F75-79 | 46.1% | 17.6% | 13.4% | 22.8% | 46.1% | | 17.4% | 13.3% | 23.2% |  |
| F80-84 | 62.2% | 9.5% | 11.3% | 17.0% | 62.2% | | 9.3% | 11.2% | 17.3% |  |
| F85+ | 52.2% | 14.1% | 11.4% | 22.2% | 52.2% | | 13.8% | 11.3% | 22.6% |  |
| Overall | 25.6% | 19.4% | 16.4% | 38.6% | 25.6% | | 18.6% | 15.8% | 40.0% |  |

MET hrs/wk, metabolic equivalent of task hours per week

Table S5. Change in modelled disease incidence by sex following the expansion of Be Active

|  | **Males** | **Females** | **Total** |
| --- | --- | --- | --- |
| Change in IHD cumulative incidence [incidence rate per 100,000] | -144 [-0.6]  (-194 to -102) | -61 [-0.3]  (-81 to -44) | -205 [-0.5]  (-275 to -147) |
| Change in stroke cumulative incidence [incidence rate per 100,000] | -305 [-1.4]  (-488 to -163) | -241 [-1.1]  (-380 to -132) | -545 [-1.2]  (-867 to -295) |
| Change in type two diabetes cumulative incidence [incidence rate per 100,000] | -2,264 [-10.5]  (-3,104 to -1,599) | -1,932 [-8.6]  (-2,631 to -1,369) | -4,199 [-9.5]  (-5,738 to -2,973) |
| Change in breast cancer cumulative incidence [incidence rate per 100,000] | 0 [0] | -125 [-0.6]  (-245 to -30) | -125 [-0.3]  (-245 to -30) |
| Change in colorectal cancer cumulative incidence [incidence rate per 100,000] | -82 [-0.4]  (-253 to 32) | -47 [-0.2]  (-143 to 22) | -130 [-0.3]  (-397 to 60) |
| Change in lung cancer cumulative incidence [incidence rate per 100,000] | 0 [0]  (0 to 1) | 0 [0]  (0 to 0) | 1 [0]  (0 to 1) |
| Change in stomach cancer cumulative incidence [incidence rate per 100,000] | 0 [0]  (0 to 0) | 0 [0]  (0 to 0) | 0 [0]  (0 to 0) |
| Change in pancreas cancer cumulative incidence [incidence rate per 100,000] | 0 [0]  (0 to 0) | 0 [0]  (0 to 0) | 0 [0]  (0 to 0)) |
| Change in kidney cancer cumulative incidence [incidence rate per 100,000] | 0 [0]  (0 to 0) | 0 [0]  (0 to 0) | 0 [0]  (0 to 0)) |
| Change in liver cancer cumulative incidence [incidence rate per 100,000] | 0 [0]  (0 to 0) | 0 [0]  (0 to 0) | 0 [0]  (0 to 0)) |
| Change in liver cirrhosis cumulative incidence [incidence rate per 100,000] | 0 [0]  (0 to 0) | 0 [0]  (0 to 0) | 0 [0]  (0 to 0)) |

Results presented are the median values from the Monte Carlo simulation, as such, the numbers in the final column may not equal the sum of males and females; 95% uncertainty intervals in parentheses; IHD, ischaemic heart disease

Table S6. Results of sensitivity analyses

| **Sensitivity analysis** | **Salt reformulation** | | **Expanding Be Active** |
| --- | --- | --- | --- |
|  | **Cost per QALY** (£) | **Return on investment**  (£ for £1 spent)* | **Cost per QALY** (£) |
| Main analysis (for reference) | Dominant | 1.44  (0.50 to 2.94) | 727,300  (513,736 to 1,063,585) |
| One year time horizon | 2,101,398  (2,516,595 to 17,345,796) | - | 12,183,276  (8,198,390 to 18,469,397) |
| Five year time horizon | 124,628  (44,612 to 388,462) | - | 1,669,123  (1,218,480 to 2,363,574) |
| 20 year time horizon | Dominant | 4.63  (1.56 to 9.52) | 312,772  (216,893 to 461,198) |
| 100 year time horizon (lifetime of cohort) | Dominant | 16.52  (5.66 to 33.31) | 91,724  (62,950 to 137,421) |
| Fall in physical activity over time | - | - | 1,052,103  (756,141 to 1,515,405) |
| No delay in the introduction of the intervention | Dominant | 1.73  (3.54 to 0.56) |  |
| No costs included from diseases not modelled by PRIMEtime CE (unrelated disease costs) | Dominant | 1.52  (0.52 to 3.05) | 735,474  (521,921 to 1,040,670) |
| Results from an NHS perspective (no social care costs) | 30,547  (13,898 to 90,823) | - | 735,851  (521,112 to 1,071,650) |
| Results from a social care perspective (no NHS costs) | Dominant | 1.21  (0.42 to 2.46) | 733,402  (519,764 to 1,069,979) |
| No industry costs included | Dominant | 110.32  (37.90 to 223.46) | - |
| Not including industry costs or government administrative costs | Dominant | 355.96  (125.38 to 743.49) | - |
| Including social care costs and productivity | Dominant | 1.57  (0.55 to 3.20) | 720,677  (512,601 to 1,047,837) |
| Including all wider societal costs | Dominant | 1.18  (0.41 to 2.41) | 705,043  (502,287 to 1,018,644) |
| Using a discount rate of 3.5% | Dominant | 1.27  (0.44 to 2.67) | 765,447  (559,808 to 1,102,006) |
| No cancer included in model | Dominant | 1.52  (0.52 to 3.05) | 784,240  (550,810 to 1,111,700) |
| Only including diseases directly related to blood pressure | Dominant | 1.41  (0.50 to 2.92) | - |
| Only including diseases directly related to physical activity | - | - | 728,128  (518,729 to 1,065,997) |
|  |  |  |  |

*Return on investment reported for analyses where the median cost per QALY is negative. There is no return on investment for the lifetime of the Be Active expansion

Median results reported from 2,000 iterations of a Monte Carlo simulation, with 95% uncertainty intervals in parentheses; QALY, quality adjusted life year

Table S7. Testing of PRIMEtime CE with extreme input values

| **Scenario** | **Change in QALYs** | **NHS savings** | **Social care savings** | **Intervention costs** |
| --- | --- | --- | --- | --- |
| Diet intervention main analysis | 15,444 | 143,566,300 | 732,265,374 | 597,785,271 |
| 0% reduction in salt consumption | 0 | 0 | 0 | 597,785,271 |
| 100% reduction in salt consumption | 81,676 | 751,842,904 | 3,984,611,598 | 597,785,271 |
| Physical activity intervention main analysis | 1,593 | 10,387,199 | 14,306,598 | 1,199,599,777 |
| 100% of the population achieve recommended physical activity levels | 169,533 | 995,815,098 | 5,552,126,423 | 1,199,653,797 |
| 100% of the population are sedentary | -231,622 | -1,467,160,814 | -3,925,887,745 | 1,199,536,675 |

All results are point estimates using mean parameter estimates and therefore main analysis results are not the same as the median results of the Monte Carlo simulation. QALY, quality adjusted life year

Table S8. Results from the UK Health Forum microsimulation model and PRIMEtime CE modelling the UK Health Forum salt reduction scenarios

| **Scenario** | **Model** | **Change in NHS costs** (£million) | **Change in IHD cumulative incidence** | **Change in stroke cumulative incidence** | **Change in IHD incidence rate/100,000** | **Change in stroke incidence rate/100,000** |
| --- | --- | --- | --- | --- | --- | --- |
| Worst case vs follows trends | UK Health Forum | 95 | 37,996 | 31,868 | 62 | 52 |
|  | PRIMEtime CE | 128  (52 to 217) | 7,553  (2,955 to 12,219) | 11,778  (4,687 to 18,910) | 14  (5 to 22) | 22  (9 to 35) |
| Best case vs follows trends | UK Health Forum | -90 | -43,512 | -34,319 | -71 | -56 |
|  | PRIMEtime CE | -663  (-1,191 to -223) | -43,529  (-71,825 to -15,075) | -59,510  (-99,747 to -20,115) | -80  (-132 to -27) | -109  (-184 to -37) |
| Expected vs follows trends | UK Health Forum | 14 | 4,903 | 9,805 | 8 | 16 |
|  | PRIMEtime CE | -65  (-201 to 42) | -5,873  (-14,095 to 1,581) | -689  (-7,883 to 6,188) | -11  (-26 to 3) | -1  (-14 to 11) |

Median values presented for PRIMEtime CE results with uncertainty intervals in parentheses; IHD, ischaemic heart disease

Table S9. Results from IMPACT CHD and PRIMEtime CE modelling the Collins et al. salt reduction scenarios[2]

| **Scenario** | **Model** | **Life years gained** (95% UI) | **NHS cost savings** (£) |
| --- | --- | --- | --- |
| Salt reduction of 20% | IMPACT CHD | 19,365  (11,967 to 27,887) | 686,572,778 |
|  | PRIMEtime CE | 10,437* | 187,694,772 |
| Salt reduction of 15% | IMPACT CHD | 14,593  (9,000 to 21,049) | 601,843,338 |
|  | PRIMEtime CE | 7,880* | 141,798,746 |
| Salt reduction of 2% | IMPACT CHD | 1,970  (1,209 to 2,854) | 434,248,212 |
|  | PRIMEtime CE | 1,069* | 19,277,616 |

*Uncertainty intervals not estimated due to computational time required to simulate each age group separately to calculate life years gained; UI, uncertainty interval

Table S10. Relative risk of disease from moving from under active or sedentary to recommended levels of physical activity used by Frew et al.[16] and PRIMEtime CE (95% uncertainty intervals)

| **Source of relative risk** | **Frew et al.** | **PRIMEtime CE** | **PRIMEtime CE** |
| --- | --- | --- | --- |
| Change in physical activity | <3 to >/=7.5 MET hrs/wk | 0 to >/=7.5 MET hrs/wk* | >0-3 to >/=7.5 METhrs/wk* |
| Ischaemic heart disease relative risk | 0.50  (0.44 to 0.56) | 0.71  (0.64 to 0.79) | 0.83  (0.78 to 0.88) |
| Stroke relative risk | 0.73  (0.67 to 0.87) | 0.72  (0.61 to 0.85) | 0.83  (0.75 to 0.91) |
| Type two diabetes relative risk | 0.57  (0.55 to 0.59) | 0.66  (0.60 to 0.74) | 0.79  (0.60 to 0.85) |
| Breast cancer relative risk | 0.81  (0.76 to 0.81) | 0.92  (0.86 to 0.97) | 0.96  (0.86 to 0.98) |
| Colorectal cancer relative risk | 0.75  (0.75 to 0.75) | 0.89  (0.72 to 1.08) | 0.94  (0.83 to 1.04) |

*Based on average physical activity levels of a 30 year old male, where sedentary is 0 MET hours/week, under active is 1.5 MET hours/week, and recommended is 28.1 MET hours/week. MET hrs/wk, metabolic equivalent of task hours per week

Table S11. Comparison of change in QALYs with a change in HALYs for the main analyses of the diet and physical activity interventions

| Scenario | Change in QALYs | Change in HALYs |
| --- | --- | --- |
| Diet intervention main analysis | 15,000  (5,928 to 25,520) | 19,531  (8,520 to 30,764) |
| Physical activity main analysis | 1,597  (1,134 to 2,169) | 1,785  (1,308 to 2,375) |

95% uncertainty intervals in parentheses. QALY, quality adjusted life year; HALY, health adjusted life year

**Additional figures**

Figure S1. Aggregate change in QALYs for each age and sex group for the salt reformulation intervention
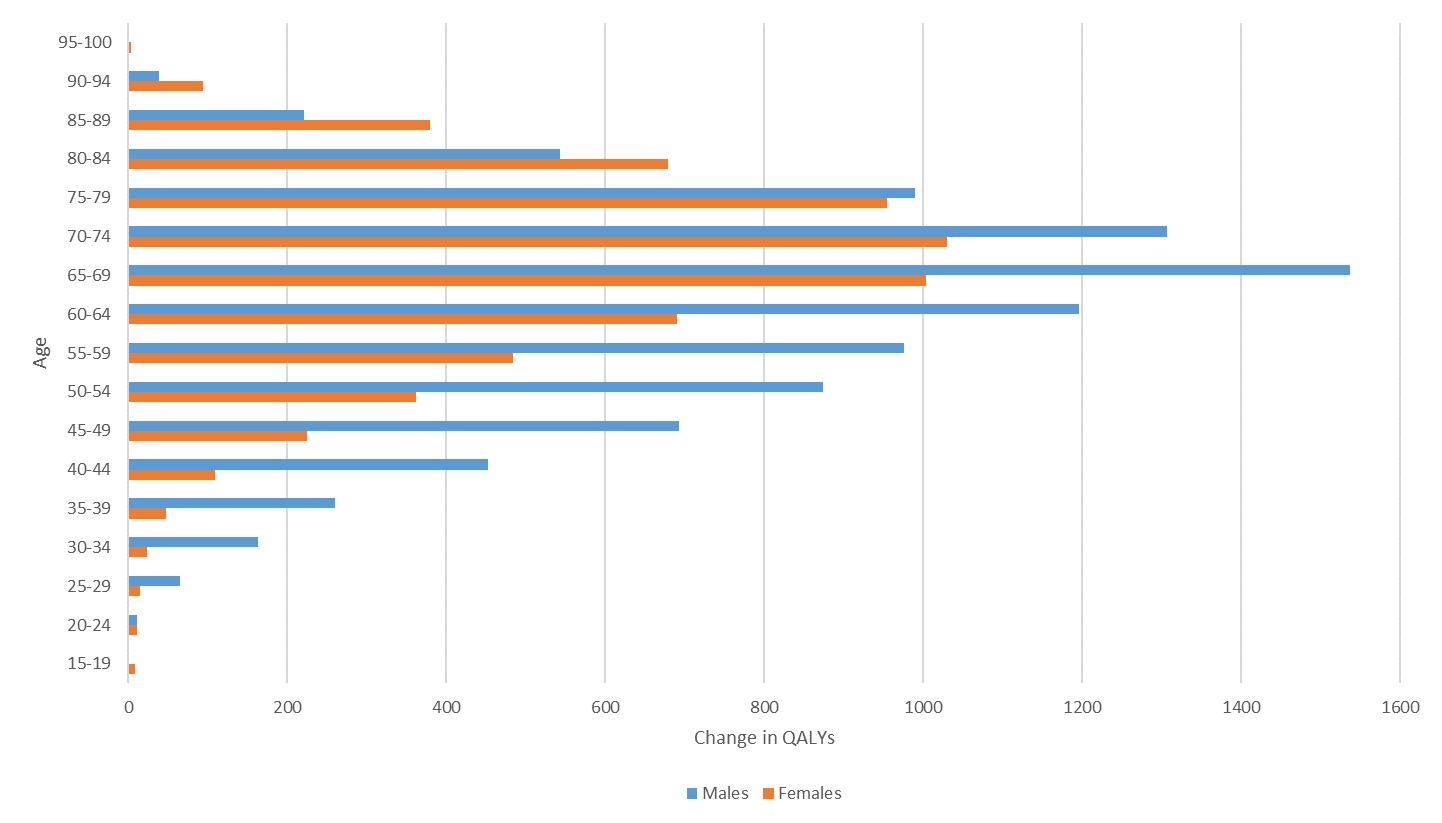


QALY, quality adjusted life year; Age specific results are based on point estimates and therefore do not include UIs.

Figure S2. Change in aggregate health and social care costs for each age and sex group for the salt reformulation intervention


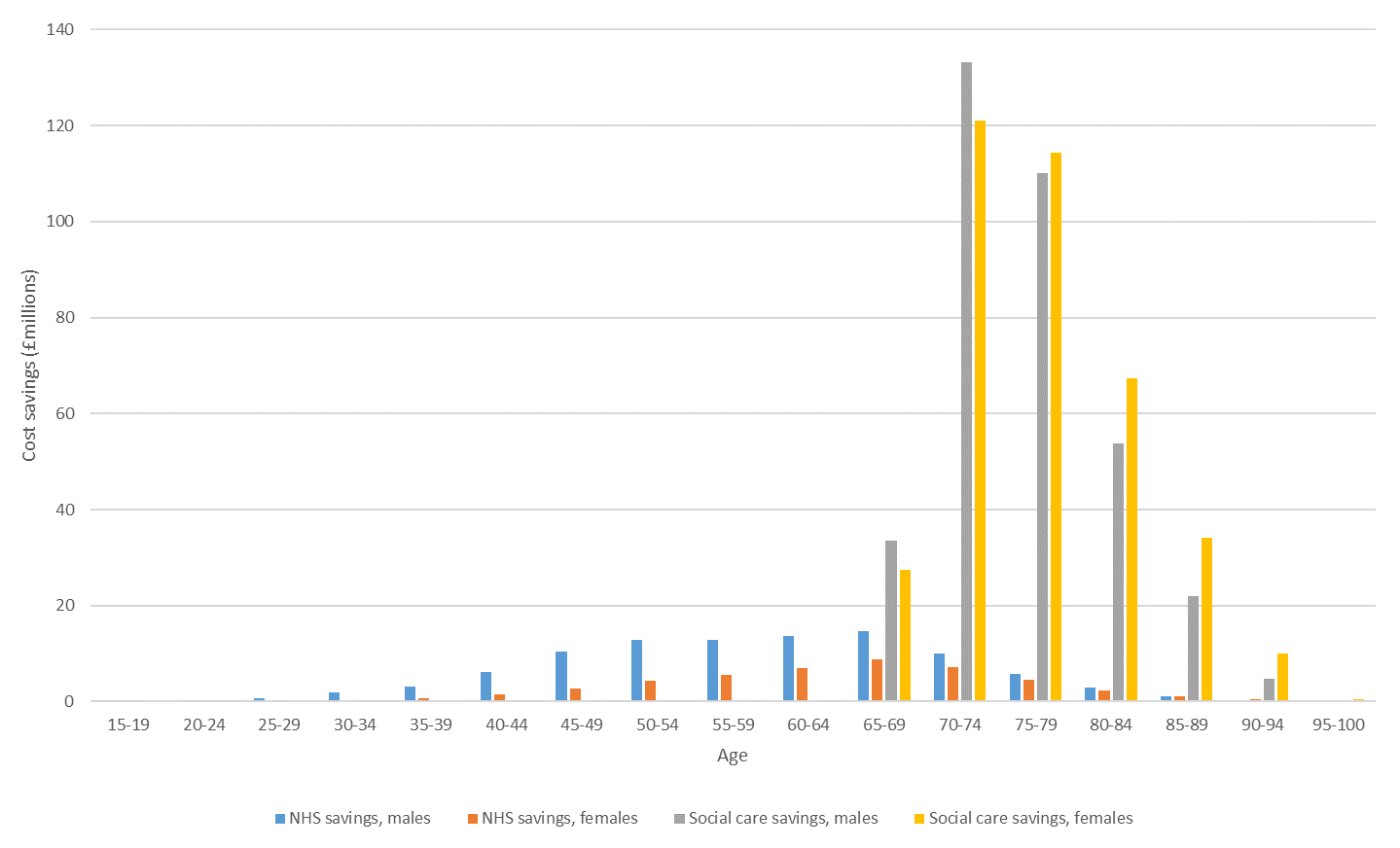
Age specific results are based on point estimates and therefore do not include UIs.

Figure S3. Aggregate change in QALYs for each age and sex group for the expansion of Be Active


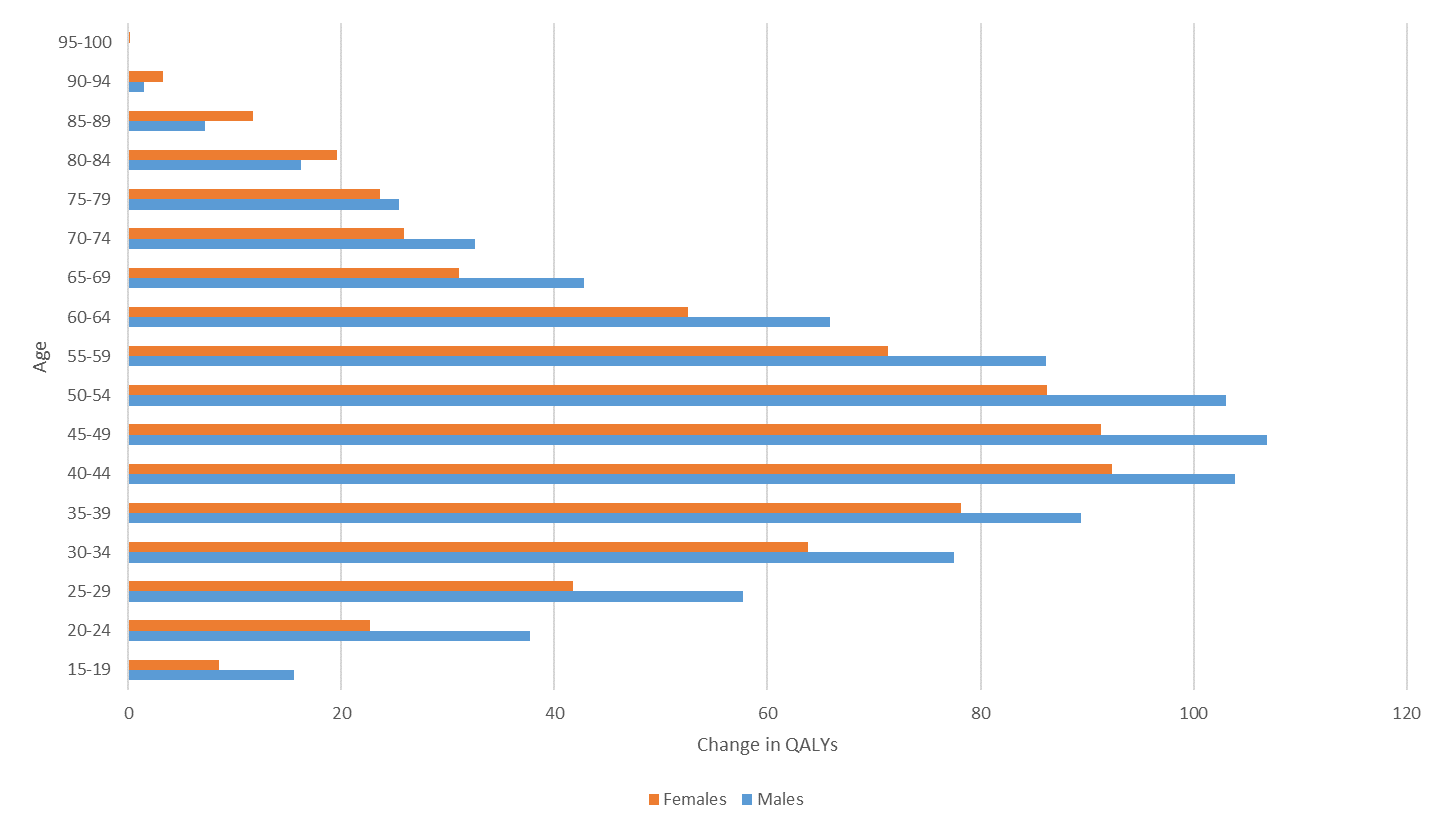


QALY, quality adjusted life year

Figure S4. Change in aggregate health and social care costs by age and sex for the expansion of Be Active


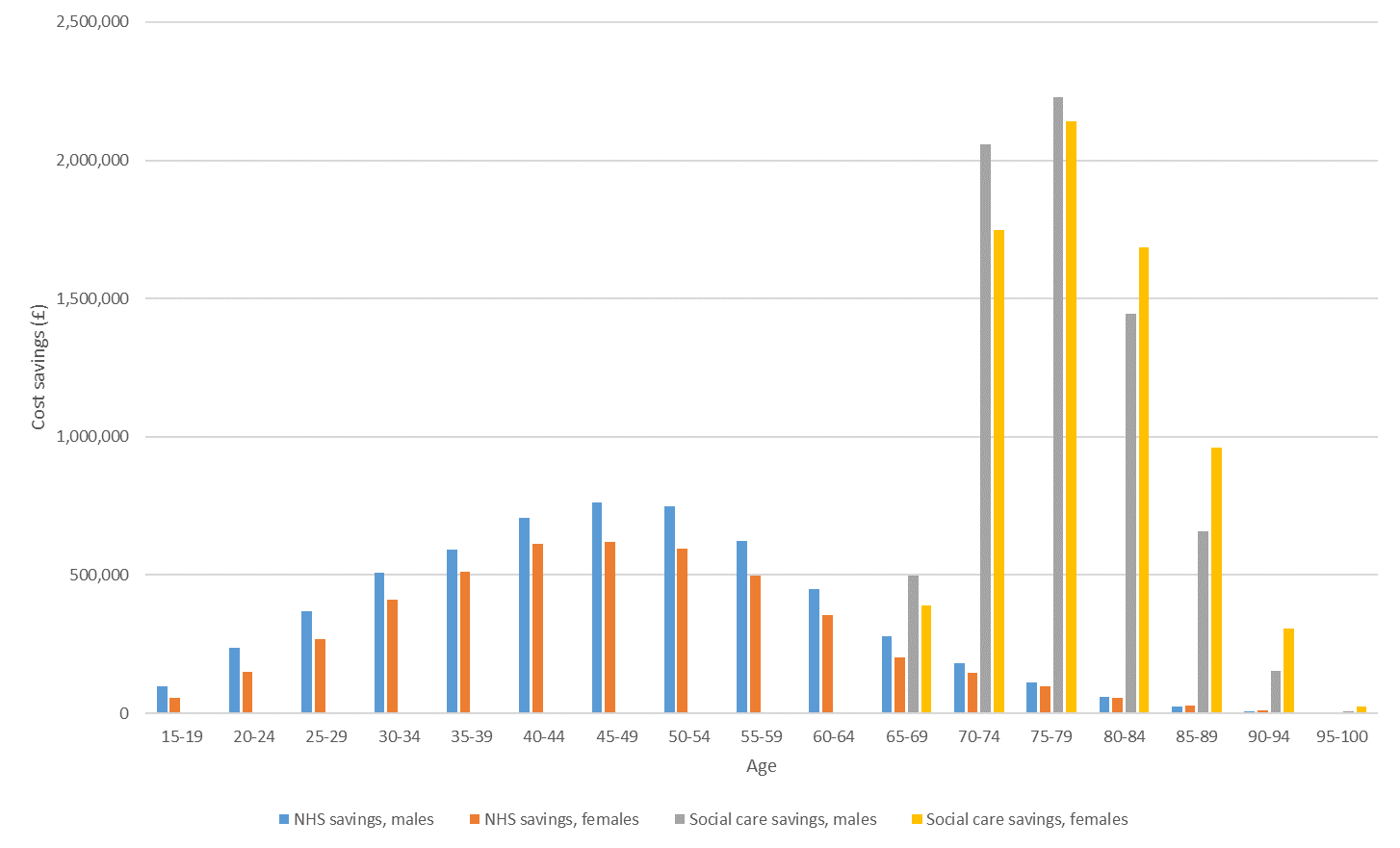


Figure S5. Cost per quality adjusted life year by age for the expansion of Be Active


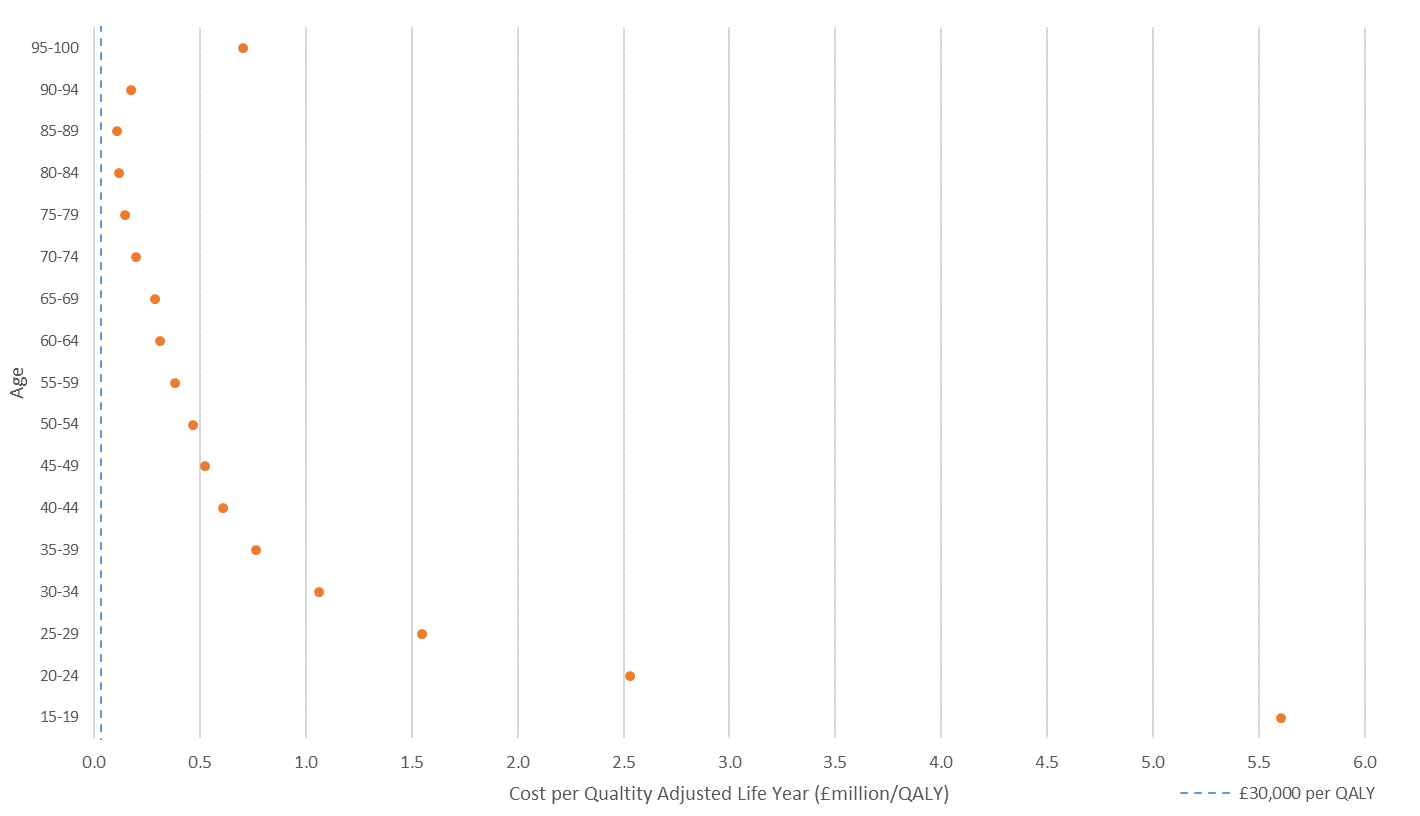
QALY, quality adjusted life year

Figure S6. Tornado plot of uncertainty of model input parameters for the salt reformulation intervention

**
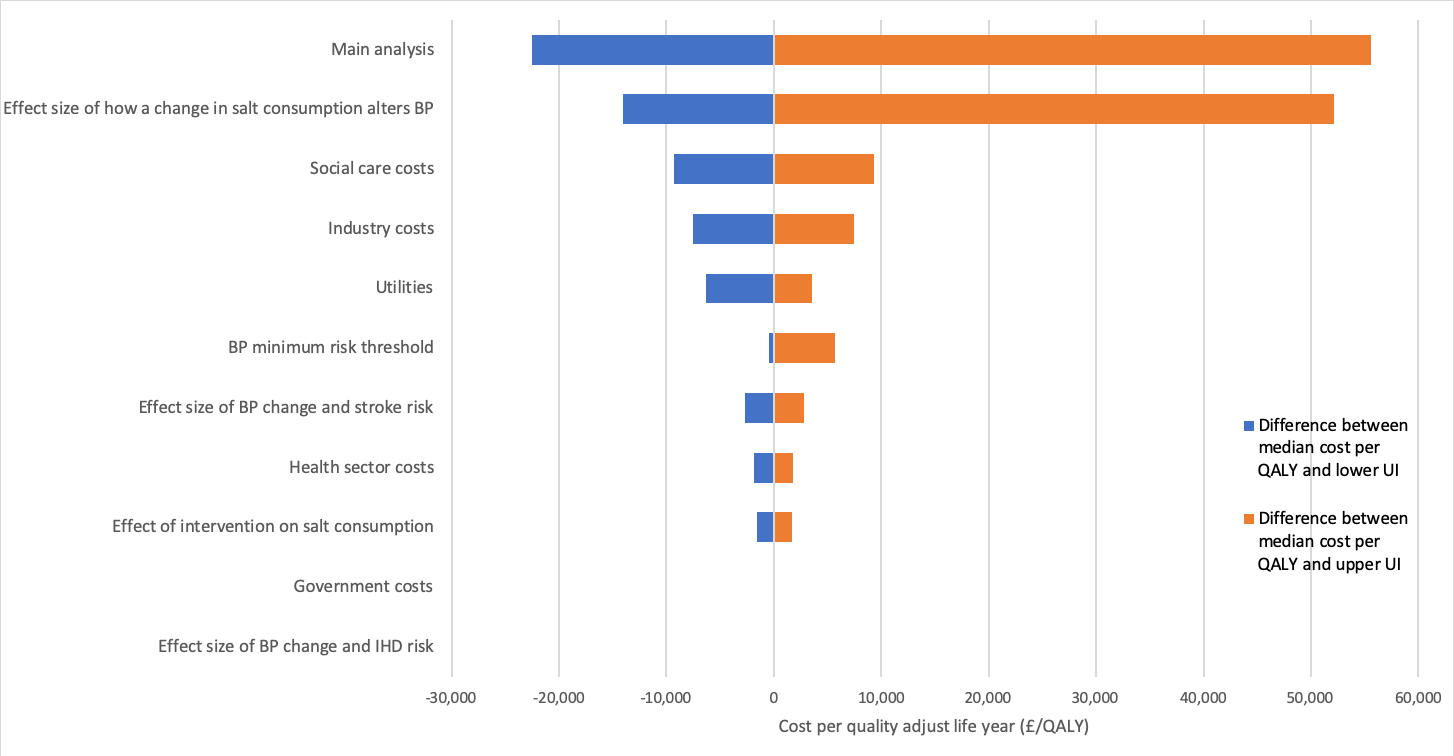
**

The bars illustrate the width of the uncertainty interval when only one parameter or collection of related parameters is allowed to vary according to their probability distribution. QALY, quality adjusted life year; UI, uncertainty interval; BP, blood pressure; IHD, ischaemic heart disease

Figure S7. Tornado plot of uncertainty of model input parameters for the expansion of Be Active


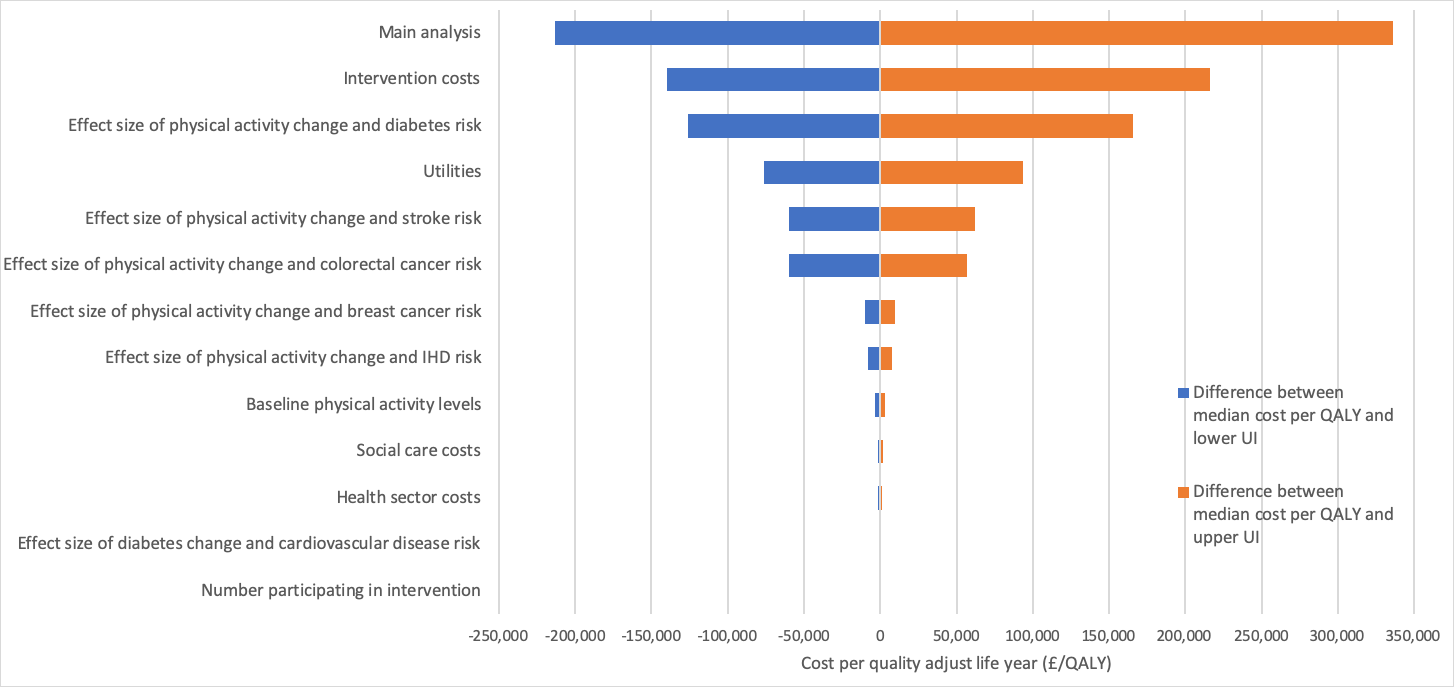


The bars illustrate the width of the uncertainty interval when only one parameter or collection of related parameters is allowed to vary according to their probability distribution. QALY, quality adjusted life year; UI, uncertainty interval; IHD, ischaemic heart disease

Figure S8 Cost effectiveness plane showing results of salt reformulation with different time horizons


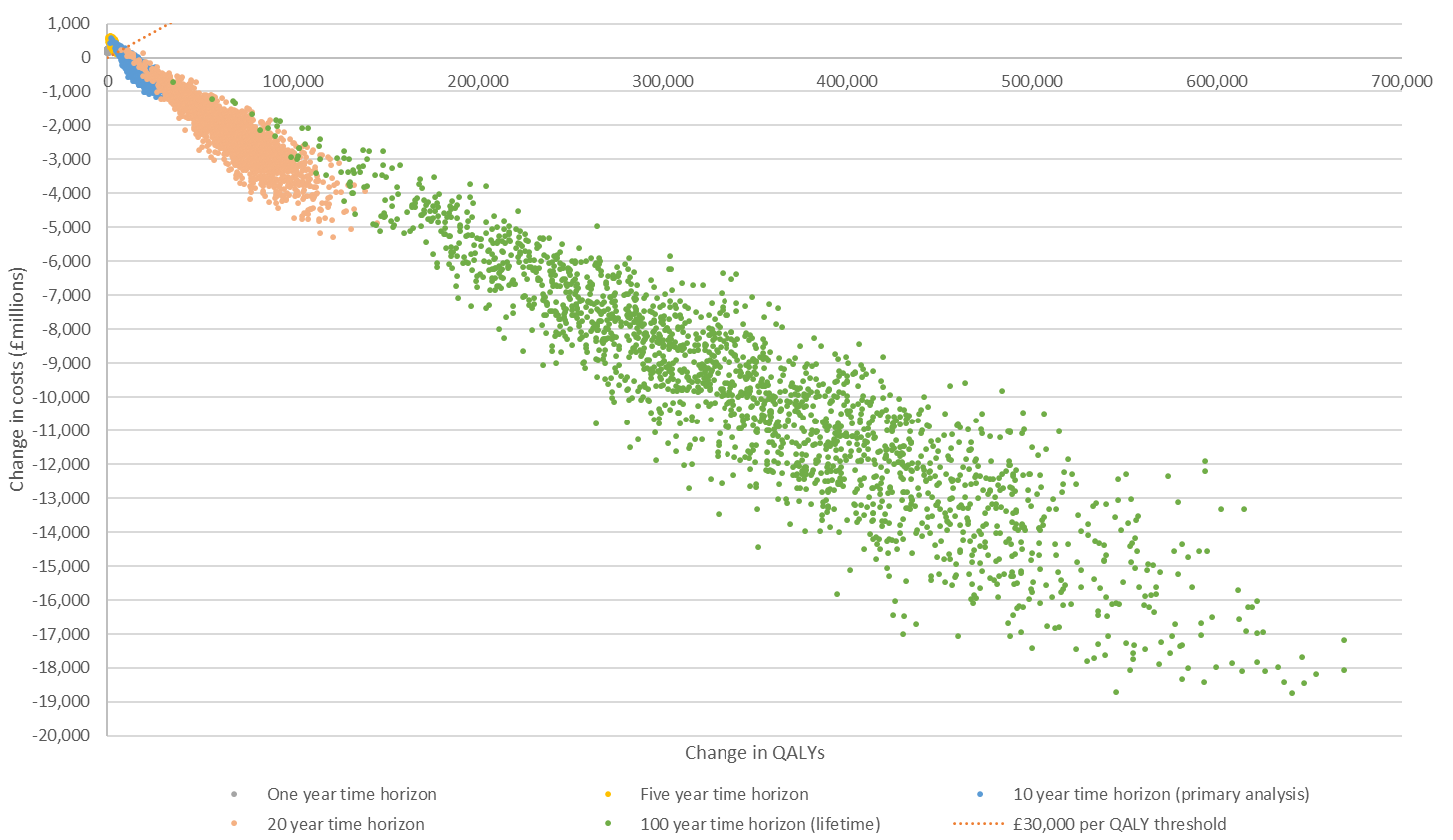


QALY, quality adjusted life year

Figure S9. Cost effectiveness plane showing results of Be Active expansion with different time horizons


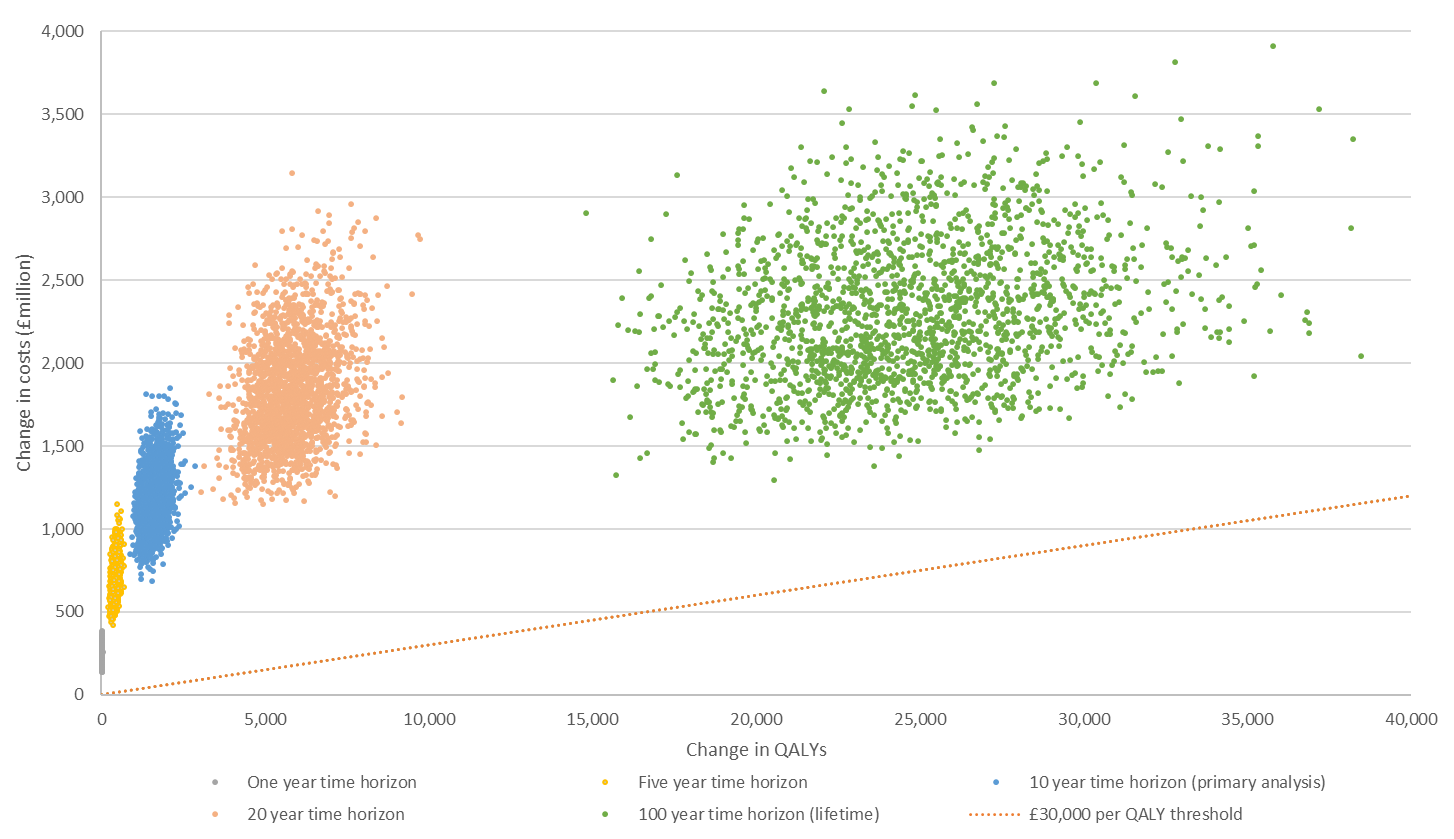


QALY, quality adjusted life year

Figure S10. Results of the UK Health Forum microsimulation model and PRIMEtime CE modelling the UK Health Forum salt reduction scenarios


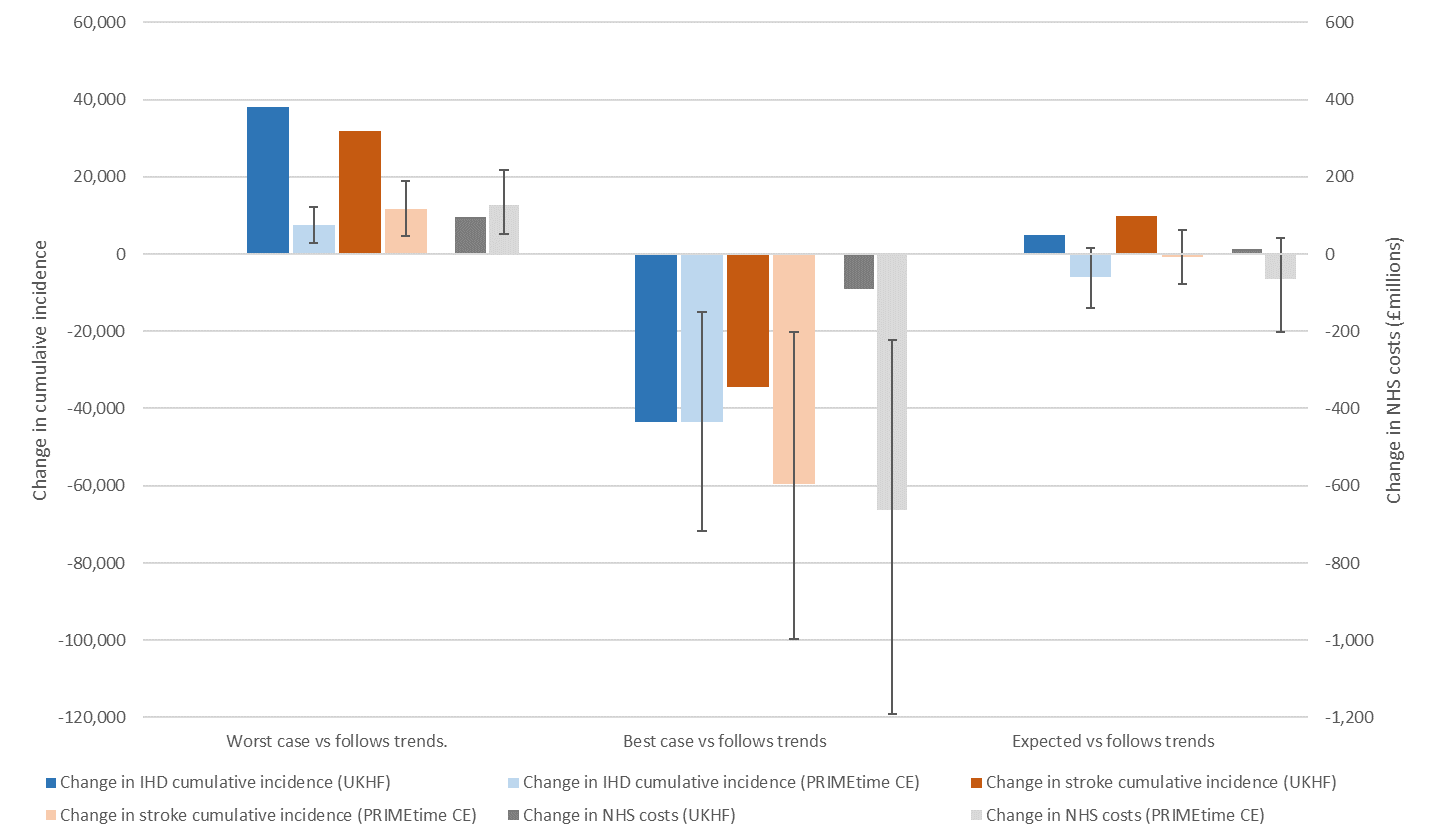


Median values presented for PRIMEtime CE results; IHD, ischaemic heart disease; UKHF, UK Health Forum

Figure S11. Results of PRIMEtime CE comparisons with Frew et al.[16]


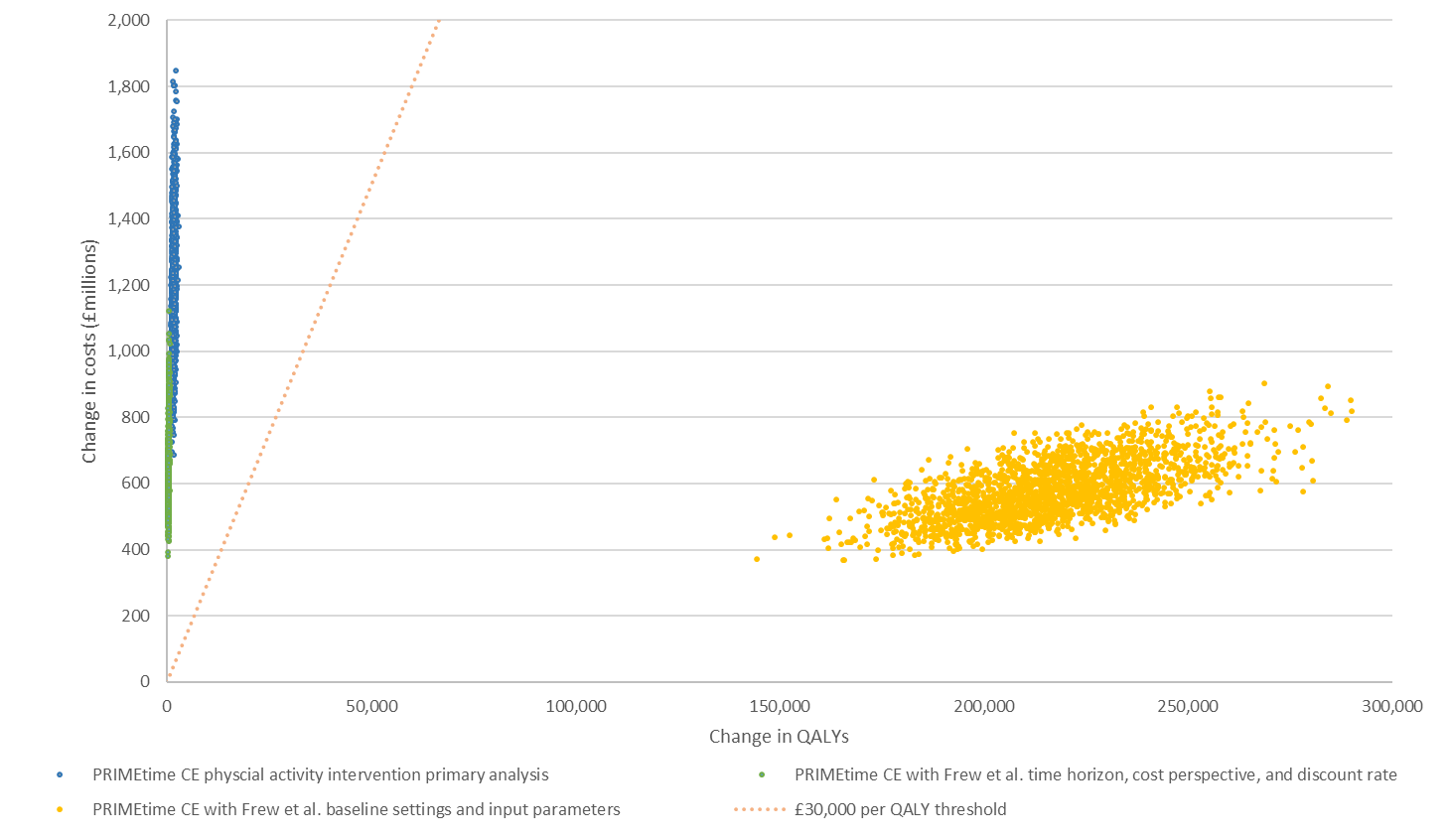


QALY, quality adjusted life year

**References**

1. NatCen Social Research, MRC Human Nutrition Research, University College London Medical School. National Diet and Nutrition Survey Years 1-4, 2008/09-2011/12. [data collection]. 7th Edition. 2015. http://dx.doi.org/10.5255/UKDA-SN-6533-6.

2. Collins M, Mason H, O’Flaherty M, Guzman-Castillo M, Critchley J, Capewell S. An economic evaluation of salt reduction policies to reduce coronary heart disease in England: a policy modeling study. Value Health. 2014;17:517–24. doi:10.1016/j.jval.2014.03.1722.

3. Food Standards Agency. Impact Assessment of the Revised Salt Reduction Targets - Annex 1. 2009.

4. Chisholm D, Mendis S, Abegunde D, Martinez RX. WHO costing tool for prevention and control of non-communicable diseases. Geneva; 2011.

5. Webb M, Fahimi S, Singh GM, Khatibzadeh S, Micha R, Powles J, et al. Cost effectiveness of a government supported policy strategy to decrease sodium intake: global analysis across 183 nations. BMJ. 2017;356:i6699. doi:10.1136/BMJ.I6699.

6. World Health Organization. Cost effectiveness and strategic planning (WHO-CHOICE). 2014. http://www.who.int/choice/cost-effectiveness/en/. Accessed 27 Feb 2019.

7. Blakely T, Cobiac LJ, Cleghorn CL, Pearson AL, van der Deen FS, Kvizhinadze G, et al. Health, Health Inequality, and Cost Impacts of Annual Increases in Tobacco Tax: Multistate Life Table Modeling in New Zealand. PLoS Med. 2015;12:e1001856. doi:10.1371/journal.pmed.1001856.

8. He FJ, Li J, MacGregor GA. Effect of longer term modest salt reduction on blood pressure: Cochrane systematic review and meta-analysis of randomised trials. BMJ. 2013;346:f1325. doi:10.1136/bmj.f1325.

9. Lewington S, Clarke R, Qizilbash N, Peto R, Collins R, Prospective Studies Collaboration. Age-specific relevance of usual blood pressure to vascular mortality: a meta-analysis of individual data for one million adults in 61 prospective studies. Lancet. 2002;360:1903–13.

10. Briggs ADM, Cobiac L, Wolstenholme J, Scarborough P. PRIMEtime CE: a multistate life table model for estimating the cost-effectiveness of interventions affecting diet and physical activity. BMC Health Serv Res. 2019;:Under Review.

11. R Core Team. R: A language and environment for statistical computing. 2016.

12. Microsoft. Microsoft Excel. 2013.

13. Sport England. Active People Survey, 2010-2011. 2013. http://dx.doi.org/10.5255/UKDA-SN-7138-3.

14. Ainsworth B, Haskell W, Herrmann S, Meckes N, Bassett D, Tudor-Locke C, et al. 2011 Compendium of Physical Activities. Med Sci Sport Exerc. 2011;43:1575–81. doi:10.1249/MSS.0b013e31821ece12.

15. Department of Health. Start Active, Stay Active: A report on physical activity from the four home countries’ Chief Medical Officers. London; 2011. https://www.gov.uk/government/uploads/system/uploads/attachment_data/file/216370/dh_128210.pdf.

16. Frew EJ, Bhatti M, Win K, Sitch A, Lyon A, Pallan M, et al. Cost-effectiveness of a community-based physical activity programme for adults (Be Active) in the UK: an economic analysis within a natural experiment. Br J Sports Med. 2014;48:207–12. doi:10.1136/bjsports-2012-091202.

17. Godin G, Shephard RJ. A simple method to assess exercise behavior in the community. Can J Appl Sport Sci. 1985;10:141–6. http://www.ncbi.nlm.nih.gov/pubmed/4053261. Accessed 22 Dec 2016.

18. Lyon A, Frew EJ, Pallan M, Park S. Evaluation of the Birmingham Be Active Programme (draft). Birmingham; 2011.

19. Office for National Statistics. Population estimates for UK, England and Wales, Scotland and Northern Ireland, revised: mid-2012, mid-2013 and mid-2014. London; 2016. https://www.ons.gov.uk/releases/populationestimatesforukenglandandwalesscotlandandnorthernirelandrevisedmid2012mid2013andmid2014.

20. Wahid A, Manek N, Nichols M, Kelly P, Foster C, Webster P, et al. Quantifying the Association Between Physical Activity and Cardiovascular Disease and Diabetes: A Systematic Review and Meta‐Analysis. J Am Heart Assoc. 2016;5:e002495. doi:10.1161/JAHA.115.002495.

21. Wahid A, Manek N, Nichols M, Kelly P, Foster C, Webster P, et al. Quantifying the association between physical activity and cancer: a systematic review and meta-analysis. 2016.

22. StataCorp. Stata statistical Software: Release 15. 2017.

23. Public Health England. National Diet and Nutrition Survey: assessment of dietary sodium. Adults (19 to 64 years) in England, 2014. London; 2016. https://www.gov.uk/government/statistics/national-diet-and-nutrition-survey-assessment-of-dietary-sodium-in-adults-in-england-2014.

24. Harper H, Hallsworth M. Counting Calories. How under-reporting can explain the apparent fall in calorie intake. London; 2016. http://www.behaviouralinsights.co.uk/publications/counting-calories-how-under-reporting-can-explain-the-apparent-fall-in-calorie-intake/.

25. Wyness LA, Butriss JL, Stanner SA. Reducing the population’s sodium intake: the UK Food Standards Agency’s salt reduction programme. Public Health Nutr. 2017;15:254–61. doi:10.1017/S1368980011000966.

26. Department for Environment Food and Rural Affairs. Family Food 2014. London; 2015. https://www.gov.uk/government/uploads/system/uploads/attachment_data/file/485982/familyfood-2014report-17dec15.pdf.

27. Health and Social Care Information Centre. Health Survey for England 2012. London; 2013. https://webarchive.nationalarchives.gov.uk/20180328130852tf_/http://content.digital.nhs.uk/catalogue/PUB13218/HSE2012-Sum-bklet.pdf/.

28. Prince SA, Adamo KB, Hamel ME, Hardt J, Connor Gorber S, Tremblay M. A comparison of direct versus self-report measures for assessing physical activity in adults: a systematic review. Int J Behav Nutr Phys Act. 2008;5:56. doi:10.1186/1479-5868-5-56.

29. Hobbs N, Godfrey A, Lara J, Errington L, Meyer TD, Rochester L, et al. Are behavioral interventions effective in increasing physical activity at 12 to 36 months in adults aged 55 to 70 years? A systematic review and meta-analysis. BMC Med. 2013;11:75. doi:10.1186/1741-7015-11-75.

30. Sport England. Active People Interactive. 2019. http://activepeople.sportengland.org/. Accessed 27 Feb 2019.

31. Mytton OT, Tainio M, Ogilvie D, Panter J, Cobiac L, Woodcock J. The modelled impact of increases in physical activity: the effect of both increased survival and reduced incidence of disease. Eur J Epidemiol. 2017;32:235–50. doi:10.1007/s10654-017-0235-1.

32. Gulliford MC, Charlton J, Bhattarai N, Charlton C, Rudisill C. Impact and cost-effectiveness of a universal strategy to promote physical activity in primary care: population-based cohort study and Markov model. Eur J Heal Econ. 2014;15:341–51. doi:10.1007/s10198-013-0477-0.

33. Moore SC, Lee I-M, Weiderpass E, Campbell PT, Sampson JN, Kitahara CM, et al. Association of Leisure-Time Physical Activity With Risk of 26 Types of Cancer in 1.44 Million Adults. JAMA Intern Med. 2016;176:816–25. doi:10.1001/jamainternmed.2016.1548.

34. Divajeva D, Retat L, Shaw A, Brown M, Webber L. Risk factor based modelling for Public Health England. London; 2014.

35. Scarborough P, Bhatnagar P, Wickramasinghe K, Smolina K, Mitchell C, Rayner M. Coronary Heart Disease Statistics 2010. London; 2010. https://www.bhf.org.uk/publications/statistics/coronary-heart-disease-statistics-2010.

36. Allender S, Peto V, Scarborough P, Boxer A, Rayner M. Coronary heart disease statistics 2006 edition. London; 2006. https://www.bhf.org.uk/publications/statistics/coronary-heart-disease-statistics-book-2006.

37. Sadler K, Nicholson S, Steer T, Gill V, Bates B, Tipping S, et al. National Diet and Nutrition Survey - Assessment of dietary sodium levels among adults (aged 19-64) in England, 2011. London; 2012. https://www.gov.uk/government/publications/assessment-of-dietary-sodium-levels-among-adults-aged-19-64-in-england-2011.

38. Cappuccio FP, Markandu ND, Carney C, Sagnella GA, MacGregor GA. Double-blind randomised trial of modest salt restriction in older people. Lancet. 1997;350:850–4. doi:10.1016/S0140-6736(97)02264-2.

39. Health and Social Care Information Centre. Hospital Episode Statistics. 2019. http://www.hscic.gov.uk/hes. Accessed 27 Feb 2019.

40. Department of Health Vascular Programme Team. Treatment of Heart Attack National Guidance. Final Report of the National Infarct Angioplasty Project (NIAP). London; 2008.

41. Medicines & Healthcare products Regulatory Authority. The Clinical Practice Research Datalink. 2017. doi:22nd June 2017.

42. Yusuf S, Hawken S, Ounpuu S, Dans T, Avezum A, Lanas F, et al. Effect of potentially modifiable risk factors associated with myocardial infarction in 52 countries (the INTERHEART study): case-control study. Lancet. 1985;364:937–52. doi:10.1016/S0140-6736(04)17018-9.
